# Structure-Preserving Excess Gibbs Learning for Multicomponent Phase Equilibria: A Falsifiable and Uncertainty-Aware Synthetic Study

**DOI:** 10.3390/ijms27198767

**Published:** 2026-09-30

**Authors:** Pedro Robles, Luis Rios-Colque, Vanesa Bazan, Luis Rojas-Valdivia

**Affiliations:** 1 Escuela de Ingeniería Química, Pontificia Universidad Católica de Valparaíso, Valparaíso 2340000, Chile; 2 Facultad de Ingeniería, Pontificia Universidad Católica de Valparaíso, Valparaíso 2340000, Chile; luis.rios.c@mail.pucv.cl; 3 Instituto de Investigaciones Mineras (IIM), Facultad de Ingeniería, Universidad Nacional de San Juan, San Juan 5400, Argentina; bazan@unsj.edu.ar; 4 Consejo Nacional de Investigaciones Científicas y Técnicas (CONICET), San Juan 5400, Argentina

**Keywords:** excess Gibbs energy, activity coefficients, phase equilibrium, scientific machine learning, Gibbs–Duhem consistency, Redlich–Kister, tangent-plane distance, uncertainty quantification, conformal prediction, reproducibility

## Abstract

This fully synthetic study tests a structure-preserving surrogate on NRTL-generated phase equilibrium systems; it contains no experimental molecular validation. A symmetric neural potential represents $G^{E}/\left(nRT\right)$, from which activity coefficients, thermal derivatives, binodals, flashes, and tangent-plane-distance diagnostics are derived. Across ten binary training seeds, its median lnγ MAE was $1.16\times 10^{-3}$, better than matched direct regression ($2.40\times 10^{-3}$) but not Redlich–Kister ($9.65\times 10^{-4}$) or correctly specified refitted NRTL ($2.36\times 10^{-4}$). Gibbs–Duhem residuals decayed as $O(h^{2})$ under grid refinement, confirming diagnostic truncation around an analytic identity. A 41,600-point binary hull gave endpoint MAE $2.31\times 10^{-4}$. On 100 ternary type-I feeds, phase-count agreement with numerical reference labels was 98.0% (exact 95% interval, 93.0–99.8%), with two false negatives after recovery of one infeasible-floor solver failure. A 20-member ensemble showed pooled error ranking (ρ=0.894) but weak in-domain ranking (ρ=0.164); a temperature–distance heuristic was stronger (ρ=0.914), and nominal 90% conformal coverage fell from 88.0% in-domain to 0.0% under shift. Controlled flashes were 2.77× slower than NRTL. A hard thermodynamic structure is therefore valuable within neural modeling, but classical dominance, shift sensitivity, and absent molecular data bound the claim.

## 1. Introduction

Phase equilibrium is a molecular-scale statement with macroscopic consequences. The same equality of chemical potentials that organizes solvent extraction, distillation, and crystallization also governs formulation stability, self-assembly, and liquid–liquid phase separation in multicomponent soft and biological matter. In computational practice, these problems are solved repeatedly inside molecular screening workflows, process simulators, and digital twins. Their numerical reliability therefore depends not only on pointwise property accuracy, but also on whether all predicted properties can be derived from a single admissible thermodynamic potential.

Classical expansions, local-composition models, and group-contribution methods—including Redlich–Kister, NRTL, UNIQUAC, UNIFAC, COSMO-SAC, and PC-SAFT—encode decades of thermodynamic knowledge and remain indispensable [1,2,3,4,5,6]. Their predictive scope is nevertheless bounded by parameter availability, functional assumptions, or quantum-chemical cost. This limitation is especially visible for emerging solvent families such as ionic liquids, for which phase equilibrium measurements and transferable parameterizations remain sparse [7,8]. Molecular machine learning has consequently progressed from matrix completion of binary interactions [9,10,11,12], through SMILES-based transformers [13], to graph neural networks that encode both intramolecular structure and intermolecular interactions [14,15].

The central difficulty is structural. A model that predicts each $\ln\gamma_{i}$ independently need not be the gradient of any scalar Gibbs energy. It may fit observations while violating Gibbs–Duhem integrability, pure-component limits, or invariance to a relabeling of species. Soft physical regularization reduces these defects but does not eliminate them [16]. Recent hard-constraint models instead learn an excess Gibbs potential and differentiate it to recover activity coefficients, thereby preserving thermodynamic identities by construction [17,18]. Related free-energy and Helmholtz-energy networks preserve Maxwell relations and equations-of-state structure [19,20]. These developments exemplify the broader transition from penalty-based physics-informed learning toward architectures whose hypothesis spaces already satisfy the governing structure [21,22].

Four gaps motivate a controlled methods study. First, hard-constraint activity-coefficient studies often emphasize binary regression; multicomponent phase topology and stability receive less attention. Second, a small pointwise error can cross a phase boundary and change the discrete number of phases. Third, pooled uncertainty metrics can be dominated by an obvious domain gap and need stratified, calibrated controls. Fourth, computational acceleration is often implied but is not automatic: a neural potential embedded in a rigorous flash may be slower than a compact analytic model. Recent work has used theory-aware surrogates as optimization warm starts [23] and introduced differentiable equilibrium operators [24]; differentiability, consistency, globality, calibration, and end-to-end speed must therefore be tested as separate claims.

This study develops and executes a deliberately bounded pilot that connects these layers (Figure 1). For the known components considered here, the model is a fitted thermodynamic surrogate, not a predictor for unseen molecules. Its contribution beyond conventional parameter fitting is the joint audit of derivative consistency, boundary conditions, species relabeling, phase decisions, and uncertainty within one computational workflow. It is not a demonstrated improvement over NRTL or UNIQUAC in chemical transferability. Indeed, the classical comparisons below delimit rather than establish a neural advantage. A shared molecular encoder is a possible extension; its structural requirements and untested chemical generalization are distinguished explicitly in Section 4.2.

The scientific question is:

What does a single invariant excess Gibbs representation guarantee, and what predictive, topological, uncertainty, and computational advantages remain after comparison with matched neural and classical baselines?

Four falsifiable hypotheses are tested:

**Hypothesis 1** **(H1).** *Hard potential learning improves held-out accuracy or differential consistency over matched direct and soft-constrained neural controls, while classical models delimit any neural claim.*

**Hypothesis 2** **(H2).** *The learned potential reproduces grid-refined binary coexistence and one-/two-phase ternary decisions, including feeds near an empirical boundary.*

**Hypothesis 3** **(H3).** *Ensemble dispersion ranks errors within domains and improves selective risk against random, temperature–distance, GP, and composition-shift controls.*

**Hypothesis 4** **(H4).** *A process-level extraction sweep preserves mass and recovery accuracy under a declared runtime protocol.*

Molecular transfer is explicitly *not* tested because the study uses fixed identity embeddings. All fitted findings are E7 numerical evidence, not experimental molecular evidence.

The contribution is fourfold: (i) an explicit contract separating identities, numerical certificates, empirical comparisons, and untested claims; (ii) a permutation-invariant pair potential with exact resident pure limits and analytic composition/temperature derivatives; (iii) a reproducible binary-to-ternary campaign with seed replication, classical baselines, resolution and stability audits, calibrated controls, and process propagation; and (iv) executable pseudocode plus a fail-closed protocol for future molecular validation. The intent is not to claim a universal molecular equation of state, but to identify precisely which conclusions follow from learned free energy in controlled systems.

## 2. Results

The results compare a learned dimensionless molar excess Gibbs energy, $g_{\theta }^{E}=G_{\theta }^{E}/\left(nRT\right)$, with direct activity-coefficient regressors and classical thermodynamic models. Here *n* is the total amount of substance, *R* the gas constant, *T* the absolute temperature, and $\gamma_{i}$ the activity coefficient of component *i*. The model definitions, disjoint data partitions, numerical tolerances, and executable pseudocodes are specified in Section 4; the formal proofs and reproducibility details are provided in Appendix A and Appendix B.

Every result in this section is E7 evidence from the archived synthetic campaign or the explicitly identified corrective numerical rerun. Classical model dominance, phase-count failures, weak within-domain uncertainty ranking, loss of conformal coverage under shift, and the runtime penalty are reported as outcomes rather than edited away.

### 2.1. Seed-Replicated Predictive Comparison Changes the Central Interpretation

Across ten independent training-noise and initialization seeds, the hard potential achieved median binary test MAE $1.16\times 10^{-3}$ (IQR $9.66\times 10^{-4}$–$1.28\times 10^{-3}$), compared with $2.40\times 10^{-3}$ for the capacity-matched direct network and $4.30\times 10^{-3}$ for the validation-selected soft-GD network (Figure 2). The paired seed-level comparison against the direct network was significant (one-sided *p* value $9.77\times 10^{-4}$). It would be incorrect, however, to infer neural superiority: the 15-parameter Redlich–Kister model reached $9.65\times 10^{-4}$, and the one-sided test favoring the hard neural potential over Redlich–Kister failed (p=0.976). Refitted fixed-α and free-α NRTL were more accurate still ($2.36\times 10^{-4}$ and $3.49\times 10^{-4}$), as expected because the teacher is NRTL. The fixed-kernel GP yielded $5.64\times 10^{-3}$, and the four-parameter UNIQUAC control yielded $6.30\times 10^{-2}$.

This result narrows H1. Hard structure is a useful neural inductive bias, but the experiment does not demonstrate that a neural pair map is preferable to a classical consistent expansion on a binary system generated by a classical model. The capacity statement was also corrected: the binary file stores 225 scalars but only 81 combinations vary or remain identifiable under the one-hot, one-pair design.

The requested hard-versus-soft comparison is therefore already quantitative: median test MAE is $1.16\times 10^{-3}$ for the hard potential versus $4.30\times 10^{-3}$ for the soft-GD MLP over the same ten training-noise seeds and data splits. At the finest independent consistency grid, their seed-0 mean absolute Gibbs–Duhem residuals are $1.07\times 10^{-7}$ and $5.12\times 10^{-3}$, respectively. The former decreases at the second order with grid spacing, whereas the latter remains nonzero. This is evidence for exact architectural consistency and better prediction in this particular comparison, not a theorem that hard constraints always lower prediction error. The soft model still improves consistency relative to unpenalized direct regression, and classical potential models remain stronger accuracy controls on the NRTL teacher.

The seed-0 property audit in Table 1 applies the same dilute, differential, and 10,400-point hull diagnostics to every comparator. Classical potential models dominate the synthetic teacher-specific quantities. For direct-output models, the reported hull uses the operational reconstruction $g^{E}=\sum_{i}x_{i}\ln\gamma_{i}$ and must not be mistaken for an integrable potential.

Figure 3 replaces the visually saturated parity plot with statewise error maps. The hard-potential residual is smooth and small over most of the domain but grows near dilute boundaries and the highest temperatures; Redlich–Kister shows the smallest of the three plotted residual fields.

The noise sweep did not support the explanation that the hard model wins only because independent label noise violates Gibbs–Duhem. The median direct/hard MAE ratio decreased from 3.00 at σ=0 to 1.70 at σ=0.05 but remained above unity at every level; its median was 1.55 under heteroscedastic noise. Learning curves and the *H*–λ validation ablation are shown in Figure 4; they expose, rather than hide, the dependence on data volume and selection.

### 2.2. Gibbs–Duhem Is a Refinement Certificate, Not a Statistical Victory

At grid sizes 301, 601, 1201, 2401, and 4801, the finite-difference residuals of analytic NRTL, Redlich–Kister, and the hard potential decayed with log–log slopes 2.00, 2.00, and 2.00, respectively (Figure 5). The hard-potential residual at the finest spacing was $1.07\times 10^{-7}$. This $O(h^{2})$ sequence demonstrates truncation error of the second-order diagnostic around a continuum identity; it is not a learned residual, and there is no “NRTL floor.” Conversely, the direct network approached a nonzero $3.55\times 10^{-2}$, and the soft-GD control approached approximately $5.1\times 10^{-3}$. Soft enforcement improved differential consistency without making it exact, which is the falsifiable contrast.

The analytic thermal derivative gave a dimensioned $H^{E}/\left(nR\right)$ MAE of 3.78 K and maximum error 19.71 K over the binary $\left(x_{1},T\right)$ plane. At 300, 350, 400, and 440 K, the mean absolute infinite-dilution error was $1.20\times 10^{-2}$ for the hard potential, $1.35\times 10^{-2}$ for the direct network, and $6.91\times 10^{-3}$ for Redlich–Kister (Figure 6). Thus, the hard boundary construction guarantees the resident pure limit but does not automatically optimize the non-resident dilute limit or the thermal derivative.

### 2.3. Binary Binodal Error Is Resolved Above the Measurement Grid

Refining the common lower-hull grid from 2600 to 10,400 and 41,600 points changed the endpoint MAE from $2.45\times 10^{-4}$ to $2.27\times 10^{-4}$ and $2.31\times 10^{-4}$, respectively. With the finest grid, the mean per-isotherm maximum composition discrepancy was $2.76\times 10^{-4}$ and the worst branch discrepancy was $1.42\times 10^{-3}$. The inverse-branch temperature MAE was 0.70 K. Because the final MAE is almost ten grid spacings at N=41,600, the discrepancy is no longer an artifact at sub-grid resolution. The 40 learned isotherms retained the same two-branch topology, although the largest error localizes near one end of the temperature range (Figure 7).

When all ensemble members were instead trained only through 370 K, the 20-member mean binodal over 390–450 K had an endpoint MAE of $4.09\times 10^{-3}$ and a maximum error of $1.04\times 10^{-2}$ (Figure 8), approximately 18 times the interpolative MAE. Endpoint dispersion and endpoint error had ρ=1.000. That perfect ordering should not be generalized: both quantities increase monotonically with the same temperature distance, so this is a topology-level confirmation of the covariate confound identified in the statewise uncertainty test.

### 2.4. Ternary Tests Expose Both Structural Value and Phase-Count Failures

Across ten ternary fits, the symmetric hard potential attained a median test MAE of $1.33\times 10^{-3}$ versus $1.99\times 10^{-3}$ for direct regression ($p=9.77\times 10^{-4}$ across seeds). The ordered-pair ablation was slightly more accurate for its single audited fit ($1.13\times 10^{-3}$) but failed joint relabeling catastrophically: its maximum cyclic-permutation residual was $6.29\times 10^{0}$ versus $4.44\times 10^{-16}$ for the symmetric potential. The direct model’s maximum permutation residual was 4.01, and its two-direction tangent Gibbs–Duhem MAE was $3.20\times 10^{-2}$. Accuracy alone therefore neither identifies nor substitutes for the structural symmetry.

On the expanded type-I panel, the numerical reference, after recovery of the failed trace feed, contains 47 one-phase and 53 two-phase labels. The learned flash returned 47 true negatives, 0 false positives, 2 false negatives, and 51 true positives: 98.0% algorithmic agreement with an exact 95% interval [93.0%, 99.8%]. The two errors occurred outside the former saturated six-feed design. Accuracy was 97.0%, 97.0%, and 100% in the near, intermediate, and interior empirical-distance terciles. Conditional tie-line and phase-fraction MAEs were $7.32\times 10^{-4}$ and $1.02\times 10^{-3}$, respectively.

The original record at zero-based index 47 failed for both solvers because its second-component feed fraction was below the imposed composition floor. The allocation-based corrective rerun resolves this failure as two-phase coexistence in both models, rather than counting the single-feed fallback as a physical answer. Each model recovered the same low-energy two-phase branch from 54 of 56 starts; the other two starts did not pass the two-phase acceptance criteria. Table 2 reports the independently checked residuals. Every additional TPD search converged; the smallest observed value was $-9.96\times 10^{-16}$, within numerical roundoff of zero. This is strong numerical evidence for the recovered candidate, not a continuum global certificate.

The corrected panel now has 100 jointly converged records and 98/100 agreement, with confusion matrix $\left[\begin{array}{cc} 47 & 0\\ 2 & 51 \end{array}\right]$ (rows: one-/two-phase reference). The aggregate percentage is unchanged because both models changed their label for the same feed. Figure 9, tie-line errors, phase-fraction errors, and distance strata use the corrected labels; the original records remain separately archived. The exact binomial interval describes agreement on this selected numerical panel, not global-solver reliability or a new chemical population. The type-II panel remains conditional on its own numerical reference and two-phase cap.

At $\mathrm{ftol}=10^{-12}$, increasing TPD starts from 18 to 100 stabilized the two nontrivial representative minima at approximately $-1.47\times 10^{-6}$ and $-3.18\times 10^{-7}$; a stable-feed test returned $1.07\times 10^{-16}$. These small negative values remain below the declared $10^{-4}$ failure threshold, but the presence of multiple local minima and imperfect recovery fractions confirms that multistart TPD is not a global certificate.

The second synthetic type-II topology produced test MAE $8.03\times 10^{-4}$ across its ten-fit campaign and 95.0% phase-count accuracy over a separate balanced 100-feed panel (95% exact interval [88.7%, 98.4%]). Figure 10 also reports the 20-feed near-boundary accuracy across all ten learned potentials. This broadens the synthetic topology test but does not add three-phase or experimental evidence.

### 2.5. Uncertainty Detects Shift but Does Not Generalize into a Coverage Guarantee

Increasing the ensemble to 20 members yielded pooled Spearman ρ=0.894 (bootstrap 95% interval [0.870, 0.914]). Stratification changed the interpretation: ρ=0.164 in-domain and ρ=0.987 under temperature shift. The zero-cost temperature–distance heuristic achieved ρ=0.914, exceeding the pooled ensemble correlation, although high-error AUROC favored the ensemble (0.998 versus 0.976). Fixed-kernel GP uncertainty had ρ=0.842.

Uncertainty-guided selection reduced AURC to 0.0020 versus 0.0076 for the mean of 200 random controls (Figure 11). Nevertheless, the nominal 90% split-conformal band covered only 88.0% of in-domain states and 0.0% under temperature shift. Split conformal provides no distribution-shift guarantee, and the observed collapse is an explicit failure result. Under the separate composition-shift experiment, pooled, central, and excluded-tail correlations were 0.597, 0.014, and 0.417. Again, most ranking signal appears between domains rather than within the supported region. H3 is therefore supported only as shift detection/selective triage, not as general epistemic calibration.

### 2.6. Process Accuracy Persists, Whereas Pointwise Runtime Remains Unfavorable

Across nine cascade configurations (4, 8, or 12 stages crossed with solvent/feed 0.25, 0.50, or 1.00), the largest recovery discrepancy was 0.193 percentage points, below the one-percentage-point falsifier; maximum component-balance error was $4.44\times 10^{-16}$. No tested configuration reached the accuracy failure threshold, so the stress test establishes only this finite operating envelope.

After three warm-ups and 30 interleaved repeats, the median reference-flash time was 0.423 s (IQR 0.362–0.464 s), compared with 1.172 s (IQR 1.087–1.273 s) for the learned potential: a 2.77× penalty (Figure 12). The potential did not require more solver work: median function evaluation counts were 945 for NRTL and 851 for the network, and median total SLSQP iterations were 209 and 189. The counters are consistent with higher per-evaluation overhead but do not replace profiling. The two methods use the same feed, solver tolerances, and start budget, with different predeclared random-start seeds; this is not a paired identical-start timing experiment. Compiled batching and warm starts remain untested engineering hypotheses.

### 2.7. External Molecular Validation Remains an Explicit Unmet Gate

No licensed experimental dataset accompanied the manuscript or reviews, and the present environment cannot establish redistribution rights or author-approved data provenance. EXP-6 was therefore not fabricated. The package contains a machine-checkable schema, manifest template, component-leakage detector, and fail-closed runner; it exits before fitting unless cited experimental rows, licence terms, frozen hashes, and component-disjoint partitions are supplied. Consequently, the revision resolves the title/scope overstatement and makes the molecular interface executable as a protocol, but experimental molecular validity remains an author-owned prerequisite for any future claim of transfer.

## 3. Discussion

### 3.1. The Defensible Contribution Is Structural, Not Neural Superiority

The expanded comparator campaign changes the scientific message in an important way. A hard excess Gibbs network outperformed matched direct and soft-penalty neural regressors, but a 15-parameter Redlich–Kister expansion was at least as accurate, and refitted NRTL was decisively better on data generated by NRTL. This is the appropriate result for a well-controlled synthetic teacher–student problem. It shows that the advantage originally attributed to the neural architecture is largely the advantage of representing a thermodynamic potential, a principle that predates machine learning [1]. Neural capacity is justified only when a chemically diverse, irregular, or high-dimensional interaction structure exceeds the reach of a compact classical expansion; that condition is not tested here.

The hard architecture nevertheless has a precise methodological value. A single scalar model makes activity coefficients, thermal derivatives, infinite-dilution behavior, common-tangent geometry, and phase-stability calculations mutually traceable. The $O(h^{2})$ Gibbs–Duhem refinement confirms that the measured residual is the truncation error of the diagnostic, while direct and soft-GD networks converge to finite violations. Pure limits and joint permutation symmetry hold for arbitrary parameter values. The ternary ordered ablation is especially informative: it fits accurately yet changes its output by order unity under a cyclic relabeling. Thus, a low MAE cannot be used as evidence that a model has learned the correct symmetry.

The binary one-hot architecture also clarifies its relation to classical solution theory. It reduces exactly to $x_{1}x_{2}\psi \left(x_{1}-x_{2},T\right)$, a nonlinear neural generalization of Redlich–Kister, with 81 identifiable rather than 225 effective parameters. This correction removes the former “smaller versus larger network” argument and replaces it with an identifiable-function comparison. Future studies should report symmetries and identifiable degrees of freedom, not just serialized weight counts.

### 3.2. From Pointwise Properties to Phase Decisions

Grid refinement separates surrogate error from numerical resolution. The fine-grid binodal discrepancy remained stable when the spacing was reduced by factors of four, and composition and inverse-temperature errors were reported independently. This supports interpolative recovery of the reference common-tangent geometry. It does not prove continuum global stability: the lower hull is exact only on its finite vertex set, and the ideal entropy term has boundary derivatives that complicate uniform Lipschitz certification on the closed simplex.

The expanded ternary campaign is more discriminating than the original six obvious two-phase feeds. It includes both classes, returns two false negatives, and attaches an exact binomial interval to the resulting 98% all-feed numerical agreement, now based on 100 jointly converged records after the trace-feed correction in Section 2.4. The finite type-II extension and ten-seed boundary panel provide a second topology. These are meaningful improvements, but the empirical opposite-class distance is only a reproducible stress coordinate; it is not the signed thermodynamic distance to the true binodal or plait point. A stronger design would construct the reference binodal explicitly, parameterize normal trajectories through it, and evaluate asymptotic phase-classification risk as the energy gap tends to zero.

The TPD saturation study is similarly informative because it exposes solver multiplicity rather than converting local optimization into a certificate by terminology. Best minima stabilized as the number of starts increased, but multiple stationary values remained, and the worst minimum was slightly negative. Deterministic global lower bounds for the small tanh potential are feasible through interval propagation, convex relaxations, or αBB/GLOPEQ-type methods [25,26]. Until such bounds are computed, the correct phrase is “multistart stability diagnostic.”

Thermal and dilute-limit tests reveal where architectural guarantees stop. The resident pure limit is exact, but the non-resident $\ln\gamma_{i}^{\infty }$ equals a learned boundary value and can be inaccurate. Likewise, analytic availability of $\partial g^{E}/\partial T$ does not make the learned enthalpy correct. The observed $H^{E}/\left(nR\right)$ and infinite-dilution errors should therefore be treated as independent validation targets in any experimental campaign, especially for solvent screening where dilute solutes dominate performance.

### 3.3. Uncertainty Results Are a Caution Against Pooled Correlations

The uncertainty audit illustrates Simpson-type aggregation. The pooled ensemble correlation is high because uncertainty and error both separate the in-domain and shifted populations; within the training support, the association is weak. A temperature–distance heuristic exceeds the pooled ensemble correlation at negligible cost. The composition-shift experiment repeats the pattern: pooled ranking is substantially stronger than central-domain ranking. Reporting only the aggregate ρ would therefore overstate the epistemic content of the ensemble.

Chemical-family and group-contribution OOD shifts have *not* been tested. A composition or temperature shift among fixed one-hot species is not a chemical holdout. Ensemble agreement provides no protection against shared structural bias: if every member predicts $f_{k}\left(s\right)=f_{\mathrm{true}}\left(s\right)+b\left(s\right)$, then $u_{s}=0$, although |b(s)| can be large. Thus the observed ranking cannot be transported to unseen chemistry. After introducing a molecular encoder, an external protocol must withhold entire chemical families or functional-group combinations, prevent molecule and mixture leakage, and report within-family ranking, error, coverage, and selective risk with group-level uncertainty intervals. Calibration must use an independent, explicitly described population; it cannot acquire an OOD guarantee merely by increasing the ensemble size. Until that protocol is executed, chemical-family robustness and calibrated molecular extrapolation remain unsupported.

Selective referral still reduces average accepted-state error and substantially improves AURC over randomized retention. This can be operationally useful as a shift detector. It is not equivalent to calibrated predictive uncertainty. The nominal split-conformal band approximately covered in-domain states but failed completely under temperature shift, illustrating that exchangeability-based coverage need not survive distribution shift, rather than a theorem that predicts zero coverage [27]. The result should discourage transporting conformal guarantees across an unmodeled distribution change. Chemically meaningful deployment requires calibration groups or covariate-shift methods that reflect molecule family, temperature, composition, and phase regime, with enough data in each stratum.

### 3.4. Molecular Interface and the IJMS Scope Boundary

The revision removes “Molecular” from the title and identifies the synthetic nature in the first abstract sentence. This is not cosmetic: canonical one-hot vectors cannot generalize to a new compound. A graph or SMILES encoder can replace the identity table without changing the scalar-potential, derivative, and equilibrium layers, but its success is an empirical question. Relevant molecular architectures already encode intra- and intermolecular interactions [13,14,15,17]; the unresolved contribution is whether their embeddings can be coupled to the present thermodynamic contract without sacrificing component-disjoint accuracy.

A credible IJMS-scale external campaign requires traceable molecules and measurements. Entire InChIKeys, mixture pairs, and systems must be isolated across train/ validation/test splits; all temperatures and compositions belonging to a held-out pair must remain outside fitting; and refitted NRTL/UNIQUAC, group-contribution, GP, direct molecular, soft-GD, and hard-potential models must receive identical observations and uncertainty weights. Binary and ternary phase endpoints, $\ln\gamma^{\infty }$, $H^{E}$, and phase count should be evaluated against measurements rather than against a fitted teacher alone. The supplied EXP-6 gate encodes these requirements but contains no fabricated or access-restricted data. Hence, the manuscript may inform molecular modeling, but it does not yet establish molecular transfer.

### 3.5. Runtime Decomposition and Process Envelope

The controlled timing result is negative but diagnostically sharper than the original single ratio. The learned flash is slower even though it takes fewer function evaluations and iterations. This is consistent with higher evaluation costs, but aggregate counters do not isolate a unique cause without function-level profiling. In the archived implementation, composition derivatives are analytic; parameter autodifferentiation is used during fitting, not repeatedly inside the flash. SLSQP receives no supplied objective Jacobian and therefore uses numerical differentiation. The scalar-energy wrapper also calls an evaluator that constructs the full analytic gradient and activity vector before discarding them, creating avoidable work. This code-path observation identifies an optimization target; it is not a measured speedup.

None of the three proposed acceleration routes—batched/compiled Jacobians, solver warm starts, or a differentiable flash—has yet demonstrated an equal-accuracy end-to-end speed recovery in this codebase. Compilation may reduce evaluation overhead; warm starts may reduce iterations but must retain independent multistart safeguards against branch locking; differentiating an equilibrium operator enables parameter learning and is not itself a guarantee of faster forward equilibrium calculations. A future benchmark must include compilation/setup and amortized timings separately, identical test states and start seeds, energy and feasibility tolerances, phase-branch agreement, failure rates, and full wall time. The current 30-repeat result remains a negative CPU benchmark, not evidence for an unimplemented accelerator.

The cascade sweep shows sub-percentage-point recovery errors over the tested stage and solvent-ratio grid, with exact algebraic balances. No tested condition reaches the one-percentage-point falsifier, so the “break point” remains outside the finite grid. Future work should approach the reference plait point, vary feed composition, and report the first certified operating condition that exceeds the tolerance. Batched compiled inference and reference-solution warm starts should then be timed end-to-end with identical convergence and failure criteria; forward-pass benchmarks alone are irrelevant to a flash workload [23,24].

### 3.6. What the NRTL Teacher Does Not Establish

The present experiments do not measure predictive performance for strongly associating fluids, electrolyte speciation, or critical endpoints. NRTL can correlate some nonideal systems phenomenologically, but an NRTL-generated benchmark cannot validate omitted molecular mechanisms. The neural model is an approximation to the chosen teacher over a finite state domain, not the discovery of a universally valid Gibbs surface. Shared smoothness can make this approximation task favorable and limits the external meaning of the observed MAEs. It does not cause the exact Gibbs–Duhem identity: that identity follows for any admissible learned scalar potential, even an inaccurate one, independently of the teacher or its labels.

These extensions require distinct physical specifications. For associating fluids, an association-aware reference such as PC-SAFT with association sites supplies a concrete non-NRTL test, accompanied by experimental phase and calorimetric data [28]. For electrolytes, the model must define ionic and apparent components, reference states, electroneutrality, and any mass-action/speciation equilibria, together with long-range electrostatic contributions [29]. A neutral-mixture activity model and its pure-species boundary conditions cannot simply be transferred to individual ions; mean ionic activities and solvent activity are appropriate measurable targets. If internal speciation variables are minimized out, derivatives must be taken consistently through that equilibrium reduction rather than fitting independent species activities.

Near a binary liquid–liquid critical point, the mean-field conditions $\partial_{x}^{2}g^{\mathrm{mix}}=0$ and $\partial_{x}^{3}g^{\mathrm{mix}}=0$ (with positive fourth derivative in the nondegenerate case) show why accurate values of $g^{E}$ or lnγ alone do not certify critical behavior. The coexistence problem becomes ill-conditioned as tie-line endpoints merge. In multicomponent mixtures, a vanishing eigenvalue of the constrained Hessian similarly signals loss of curvature, but a critical endpoint requires its own phase-topology analysis. No critical location, exponent, or near-critical error scaling is validated here. A finite smooth tanh model is not evidence of asymptotic critical scaling. A convincing extension must test higher derivatives, coexistence branches, solver conditioning, and model discrepancy against independent data and physics-aware baselines. These are prospective tests, not additional outcomes of this NRTL campaign.

### 3.7. Limitations and Claim Status

The remaining limitations are substantive:

1. **Synthetic circularity.** NRTL is both a data generator and an evaluation reference. The campaign measures approximation and numerical propagation, not model discrepancy with nature.
2. **No molecular transfer.** One-hot identities contain no chemical structure; EXP-6 awaits author-supplied, licensed experimental data.
3. **Restricted topology.** Both ternary calculations restrict the search to at most two phases; this is not a certified exclusion of higher phase counts. No type-III region, critical endpoint, three-phase triangle, VLE, VLLE, solid, reactive, or electrolyte equilibrium is tested.
4. **Finite/local stability.** Binary globality is grid-relative and ternary TPD is multistart local optimization.
5. **Derivative sensitivity.** Hard consistency ensures relationships among learned derivatives, not the experimental accuracy of thermal or dilute-limit properties.
6. **Shift-dependent uncertainty.** Within-domain rankings are weak and nominal conformal coverage does not survive distribution shift.
7. **Implementation-bound timing.** Results apply to the archived CPU, single-threaded, pointwise Python implementation; compiled/warm-start performance remains unknown.
8. **Author-owned metadata.** Dataset DOI, repository DOI, funding, conflicts, final CRediT roles, and submission dates cannot be scientifically inferred and must be confirmed by the authors.

Classical thermodynamic identities and reference equations are established knowledge (E1); formal consequences in Appendix A are derived results (E6); the architecture and workflow are methodological proposals (E4); and every fitted metric is E7. Experimental molecular validity, global continuum certification, calibrated OOD coverage, and industrial acceleration remain unachieved claims.

## 4. Materials and Methods

This computational study uses the archived software versions and execution environment documented in Appendix B, including Python 3.12.13, NumPy 2.3.5, SciPy 1.17.0, scikit-learn 1.8.0, Matplotlib 3.10.8, and Autograd 1.8.0.

### 4.1. Thermodynamic State Space, Scope, and Consistency Requirements

The term *epistemic contract*, used in the earlier presentation, means a transparent separation between what is guaranteed mathematically, what is checked numerically, and what is supported by data. It is not an additional chemical law. We use *thermodynamic consistency requirements* for the architectural conditions below. For a chemist, the distinction is that Gibbs–Duhem can hold exactly while the predicted activity coefficients are inaccurate: consistency is necessary for an admissible model but is not evidence of agreement with measurements. The evidence labels retained for traceability mean established thermodynamics (E1), a proposed method (E4), a mathematical derivation (E6), and a result of the present synthetic numerical experiment (E7); they are not an external evidence-grading standard.

Let $N_{c}$ be the number of components, $n\in R_{+}^{N_{c}}$ the mole vector, $n=\sum_{i}n_{i}$, and

(1) $$\Delta^{N_{c}-1}=\left\{x\in R_{+}^{N_{c}}:\sum_{i=1}^{N_{c}}x_{i}=1\right\},\;x=n/n,$$

the composition simplex. At a fixed pressure, the modeled dimensionless molar mixing Gibbs energy is

(2) $$g_{\theta }^{\mathrm{mix}}\left(T,x\right)=\sum_{i=1}^{N_{c}}x_{i}\ln x_{i}+g_{\theta }^{E}\left(T,x\right),$$

where $g_{\theta }^{E}=G_{\theta }^{E}/\left(nRT\right)$. Its extensive counterpart is $G_{\theta }=nRTg_{\theta }^{\mathrm{mix}}$. The study is deliberately restricted to low-to-moderate-pressure liquid–liquid equilibrium; pressure-dependent and compressible systems require a Helmholtz-energy or equation-of-state extension.

All numerical evidence is synthetic NRTL evidence (E7). Canonical one-hot identity vectors distinguish the components but contain no molecular graph or descriptor information. Accordingly, this work tests a thermodynamic representation and its equilibrium consequences; it does not test chemical transfer, unseen molecules, or agreement with experiment. No experimental row has been invented to satisfy journal scope. The archive instead supplies a provenance-checked EXP-6 protocol that remains disabled until the authors provide licensed, cited measurements and a frozen component-disjoint split.

Table 3 separates exact architecture/solver properties from numerical or unresolved statements.

Homogeneity of $G_{\theta }$ gives the ambient-coordinate expression

(3) $$\ln\gamma_{i}=g_{\theta }^{E}+\frac{\partial g_{\theta }^{E}}{\partial x_{i}}-\sum_{j=1}^{N_{c}}x_{j}\frac{\partial g_{\theta }^{E}}{\partial x_{j}}.$$

The apparent ambient-extension ambiguity cancels because gradients of two extensions that agree on the simplex differ only along 1, which Equation (3) annihilates (Appendix A). Gibbs–Duhem consistency, $\sum_{i}x_{i}\;d\ln\gamma_{i}=0$ at fixed T,P, is consequently an analytic identity of the representation, not an empirical hypothesis.

### 4.2. Symmetric Excess Gibbs Architecture and Identifiability

Each component has an identity embedding $h_{i}\in R^{3}$. The learned potential is a shared sum over unordered pairs,

(4) $$g_{\theta }^{E}\left(T,x\right)=\sum_{1\leq i<j\leq N_{c}}x_{i}x_{j}\;\psi_{\theta }\;\left(\phi_{ij}\right),$$

with

(5) $$\phi_{ij}=\left[h_{i}+h_{j},h_{i}⊙h_{j},\left(x_{i}-x_{j}\right)\left(h_{i}-h_{j}\right),x_{i}+x_{j},x_{i}x_{j},\tilde{T}\right],\;\tilde{T}=\frac{T-T_{c}}{T_{s}},$$

and

(6) $$\psi_{\theta }\left(\phi \right)=a^{T}\tanh\left(W\phi +b\right)+c.$$

The binary scaling is $T_{c}=370$ K, $T_{s}=80$ K; the isothermal ternary scaling is $T_{c}=323.15$ K, $T_{s}=50$ K. Pair exchange leaves every feature unchanged, and a joint component permutation merely reindexes the unordered sum. The $x_{i}x_{j}$ multiplier gives exact resident pure-component limits, while the absent-component limit remains finite and trainable; Appendix A shows $\ln\gamma_{i}^{\infty }=\psi_{ik}$ when *i* is diluted into pure *k*.

For *H* hidden units and feature width *F*, the stored layout is row-major $\left[\mathrm{vec}\left(W\right)\in R^{HF},b\in R^{H},a\in R^{H},c\right]$. The binary H=16 file therefore contains 225 scalars. This is not its effective capacity: with one unordered one-hot pair, $h_{i}+h_{j}$ and $x_{i}+x_{j}$ are constant and $h_{i}⊙h_{j}=0$. The model reduces exactly to the neural Redlich–Kister form

(7) $$g_{\theta }^{E}=x_{1}x_{2}\;\psi_{\theta }\left(x_{1}-x_{2},\tilde{T}\right),$$

with 81 identifiable scalar combinations (three varying input weights plus one effective bias for each hidden unit, 16 output weights, and one output bias). The manuscript therefore reports both 225 stored and 81 identifiable parameters and does not use raw parameter count as evidence of a conservative comparison. The ternary model uses H=20; uncertainty members use H=12, both declared in the frozen configuration.

A molecular extension would replace only the identity table by

(8) $$h_{i}=E_{\eta }\left(G_{i}\right)\in R^{d},\;E_{\eta }\left(QG_{i}\right)=E_{\eta }\left(G_{i}\right),\;F=3d+3,$$

where $G_{i}$ is a molecular graph and *Q* relabels its atoms. A shared message-passing encoder with an atom-permutation-invariant readout can satisfy this condition. Equation (5) retains exactly the same sums, products, and signed composition–embedding difference; only the embedding width and first-layer width change. The factor $x_{i}x_{j}$ remains unchanged. Finite embeddings and a pair map with bounded first composition derivatives near each vertex preserve the resident pure limit. A twice continuously differentiable composition map supplies the classical Gibbs–Duhem identity.

The guarantee is independent of the encoder’s internal message-passing design only *conditional on* atom-label invariance, shared species-wise encoding, symmetric pair features, and differentiation of the complete scalar potential. These are checkable assumptions, not properties of every network called a GNN. If embeddings depend on mixture composition through intermolecular message passing, the encoder must also be equivariant under species relabeling and its composition dependence must be included in the chain rule; treating such embeddings as constants generally invalidates Equation (3) as implemented. Proposition A3 makes the scope precise. Untrained abstract-graph unit tests verify this interface, not molecular prediction.

Related molecular potential models establish the feasibility of this design direction [17,18]. However, a new encoder must be trained across chemically diverse systems and tested on entirely held-out molecules, pairs, scaffolds, and chemical families, with hyperparameters fixed before test disclosure. The present one-hot fits provide no such transfer result. The supplied experimental schema checks provenance and component leakage; a family/group-contribution holdout remains a separate, unexecuted validation requirement.

### 4.3. Synthetic Reference Systems and Disjoint Data Splits

The analytic NRTL reference is [2]

(9) $$g_{\mathrm{NRTL}}^{E}=\sum_{i=1}^{N_{c}}x_{i}\frac{\sum_{j=1}^{N_{c}}x_{j}\tau_{ji}G_{ji}}{\sum_{k=1}^{N_{c}}x_{k}G_{ki}},\;G_{ji}=\exp\left(-\alpha_{ji}\tau_{ji}\right),$$

and its composition gradient is analytic (Appendix A). The binary system has $\tau_{12}=\tau_{21}=850/T$ and $\alpha_{12}=\alpha_{21}=0.20$. The ternary type-I system at 323.15 K uses $\left(\tau_{12},\tau_{21}\right)=\left(0.45,0.30\right)$, $\left(\tau_{13},\tau_{31}\right)=\left(3.15,3.05\right)$, $\left(\tau_{23},\tau_{32}\right)=\left(0.55,0.70\right)$, and $\alpha_{ij}=0.20$. These are synthetic topology generators, not parameters of the illustrative component-role labels.

Binary compositions were uniform on $x_{1}\in \left[0.004,0.996\right]$ and T∈[290,450] K; mutually disjoint training, validation, and noise-free test sets contained 420, 240, and 2400 states. Ternary training, validation, and test sets contained 720, 240, and 1800 Dirichlet samples (concentration 0.85). Training labels received independent normal perturbations with σ=0.01 (binary) or 0.006 (ternary). Such componentwise perturbations need not satisfy Gibbs–Duhem and therefore model label noise rather than a realizable thermodynamic field. To expose any structural advantage induced by this choice, the revision repeats the binary study for σ∈{0,0.005,0.01,0.02,0.05} and for heteroscedastic σ=0.003+0.006|lnγ|.

### 4.4. Training, Model Selection, and Comparator Family

The hard potential minimizes

(10) $$L_{\mathrm{pot}}\left(\theta \right)=\frac{1}{\left|D\right|N_{c}}\sum_{s\in D}{∥\ln\gamma_{\theta ,s}-y_{s}∥}_{2}^{2}+\lambda \frac{{∥\theta ∥}_{2}^{2}}{n_{\theta }}.$$

L-BFGS-B uses automatic parameter gradients, maximum 650–800 iterations, function tolerance $10^{-9}$, gradient tolerance $10^{-6}$, and at most 40 line-search steps. Candidate $\lambda \in \{0,10^{-9},3\times 10^{-8},10^{-7}\}$ and soft-GD weights $\left\{10^{-3},10^{-2},10^{-1},1\right\}$ were selected once on validation data, then frozen across test evaluation and ten independent noise/initialization seeds.

The binary comparator family comprises: (i) a matched 82-parameter tanh network predicting both $\ln\gamma_{i}$ directly; (ii) the same network with a differentiable Gibbs–Duhem penalty [16]; (iii) a 15-parameter Redlich–Kister expansion with five composition and three temperature terms [1]; (iv) NRTL refitted with fixed α=0.20; (v) NRTL with fitted α; (vi) a four-parameter binary UNIQUAC control with fitted size/surface and interaction terms [3]; and (vii) a fixed-kernel Gaussian process [30]. Identical observations, splits, noise seeds, and test metrics were used. Because the synthetic teacher belongs to the NRTL family, refitted NRTL is expected to be a stringent or dominant comparator; this is a feature of the audit, not an unfavorable baseline to suppress.

For ternary data, ten hard-potential and direct-network fits were compared on the same test states. A deliberately order-sensitive pair ablation replaced symmetric features by $\left[h_{i},h_{j},x_{i},x_{j},\tilde{T}\right]$. Joint cyclic permutation was evaluated for all three models, and two independent simplex-tangent Gibbs–Duhem residuals were computed for the direct network. Statistical tests use the ten independent fitted procedures as replicates. State-level pseudo-replication and the former 2400-state Wilcoxon test were removed.

For reproducibility, the soft binary comparator predicts $f_{\omega }=\left(f_{1},f_{2}\right)=\ln\gamma$ and minimizes

(11) $$\begin{array}{cc} L_{\mathrm{soft}}= & \frac{1}{2N}\sum_{s=1}^{N}∥f_{\omega }\left(x_{s},T_{s}\right)-y_{s}{∥}_{2}^{2}+\frac{\lambda }{n_{\omega }}{∥\omega ∥}_{2}^{2}\\ \; & +\frac{\lambda_{\mathrm{GD}}}{N}\sum_{s=1}^{N}{\left[x_{s}\frac{\partial f_{1}}{\partial x_{1}}+\left(1-x_{s}\right)\frac{\partial f_{2}}{\partial x_{1}}\right]}_{\left(x_{s},T_{s}\right)}^{2}. \end{array}$$

Here $x_{s}$ denotes the mole fraction of component 1, and derivatives follow the binary simplex path $x_{2}=1-x_{1}$. They are evaluated analytically for the tanh MLP; automatic differentiation supplies parameter gradients during training. Setting $\lambda_{\mathrm{GD}}=0$ gives the matched direct control. The validation-selected value is $\lambda_{\mathrm{GD}}=1$; both direct-output controls use $\lambda =10^{-7}$, whereas the selected hard-potential regularizer is $10^{-9}$. A finite penalty acts at sampled states and does not impose an identity throughout the composition domain. The predictive and consistency comparisons are therefore reported separately, and superiority is restricted to the measured neural-control comparison rather than asserted for all models or fluids.

Data efficiency was audited at $N_{\mathrm{train}}\in \left\{105,210,420,840,1680\right\}$ across ten seeds. A 4×4 ablation crossed H∈{8,16,24,32} with the four regularization values. No test observations participated in these selections.

### 4.5. Composition and Thermal Derivatives

Equations (4)–(6) are differentiated analytically with respect to each ambient composition coordinate. Automatic differentiation is confined to optimization with respect to parameters. Because temperature occurs in the final input coordinate,

(12) $$\frac{H^{E}}{nR}=-T^{2}\frac{\partial g_{\theta }^{E}}{\partial T},$$

is also evaluated analytically and checked against a centered-temperature derivative. Infinite-dilution activity coefficients were evaluated at $x_{i}=10^{-8}$ at 300, 350, 400, and 440 K. Permanent tests compare the thermal derivative to finite differences and the analytic NRTL limits to independently fixed values.

Gibbs–Duhem residuals were evaluated using the same centered second-order composition stencil at N∈{301,601,1201,2401,4801}. The reference, hard potential, and Redlich–Kister residuals are expected to decrease as $O(h^{2})$ because their continuum residual is exactly zero; this is a numerical certificate of differentiation error. The matched direct and soft-GD networks are the falsifiable consistency controls. The term “NRTL floor” is not used.

### 4.6. Binary Coexistence and Resolution Audit

For each of 40 temperatures from 292 to 448 K, the largest interval skipped by the one-dimensional lower convex hull of Equation (2) defines the discrete binodal. The reference and surrogate use the identical operator. The computation was repeated on 2600, 10,400, and 41,600 uniformly spaced points. Endpoints are grid vertices; no interpolation is implied. The revision therefore reports grid spacing, left/right endpoint residuals, mean and maximum composition errors, and an independently interpretable inverse-branch temperature MAE. The former mixed-axis Hausdorff scalar is retained only in the historical archive, not as the primary metric. The finite hull is globally optimal over its sampled vertices but is not a continuum certificate.

### 4.7. Ternary Flash, Phase Classification, and Stability

With $N_{p}^{\max}=2$ (the maximum phase count admitted by this benchmark), equilibrium minimizes

(13) $$\min_{\beta ,x^{A},x^{B}}\beta g^{\mathrm{mix}}\left(x^{A}\right)+\left(1-\beta \right)g^{\mathrm{mix}}\left(x^{B}\right)\;\mathrm{subject}\;\mathrm{to}\;\beta x^{A}+\left(1-\beta \right)x^{B}=z.$$

Phase B is eliminated algebraically, leaving β and two coordinates of phase A. Four deterministic plus ten random starts are optimized by SLSQP with $\mathrm{ftol}=10^{-11}$ and at most 600 iterations. The single-feed energy competes with every feasible split; two phases require energy gain $>2\times 10^{-8}$ and $\ell_{1}$ phase separation $>2\times 10^{-3}$. Function evaluations, iterations, successful starts, energy gain, and mass reconstruction are archived.

The revised type-I panel contains 100 feeds: 50 prescreen-labeled reference-one-phase and 50 reference-two-phase states selected from a prescreened simplex sample. Its empirical boundary coordinate is the Euclidean distance to the nearest sampled feed of the opposite reference class. This is not a thermodynamic signed-distance theorem; it is a reproducible stratification variable defined for both classes. Phase-count results are reported as a confusion matrix, a two-sided 95% Clopper–Pearson interval [31], and accuracy in near/intermediate/interior distance terciles. Tie-line and phase-fraction errors are conditional on both solvers returning two phases. The benchmark searches only one- and two-phase candidates; this imposed cap is not proof that a third phase is globally impossible.

The convergence audit exposed an infeasible numerical floor, not a failure of the physical balance equations: imposing $x_{i}^{A},x_{i}^{B}\geq \epsilon$ forces $z_{i}\geq \epsilon$. The original $\epsilon =2\times 10^{-6}$ therefore excluded a feed with $z_{2}=7.8033572665\times 10^{-8}$. We recovered the failed record without clipping the feed or refitting either model, using component-allocation variables $0<r_{i}<1$ per unit total feed:

(14) $$n_{i}^{A}=z_{i}r_{i},\;n_{i}^{B}=z_{i}\left(1-r_{i}\right),\;\beta =\sum_{i}n_{i}^{A},\;x_{i}^{A}=\frac{n_{i}^{A}}{\beta },\;x_{i}^{B}=\frac{n_{i}^{B}}{1-\beta }.$$

These variables preserve positivity and every component balance algebraically. For the dimensionless total mixing energy $F=\beta g\left(x^{A}\right)+\left(1-\beta \right)g\left(x^{B}\right)$, extensivity gives $\partial F/\partial r_{i}=z_{i}\left(m_{i}^{A}-m_{i}^{B}\right)$, where $m_{i}=\ln x_{i}+\ln\gamma_{i}$. Twenty-four deterministic allocations and 32 fixed-seed random allocations were minimized by bounded L-BFGS-B, followed by bounded least-squares refinement of $m_{i}^{A}-m_{i}^{B}=0$. The refinement removes the small $z_{i}$ scaling that can hide trace-component disequilibrium in the objective gradient. Allocation bounds were $\left[10^{-10},1-10^{-10}\right]$; accepted candidates required chemical-potential residual $\leq 10^{-8}$, balance residual $\leq 10^{-12}$, positive compositions, and the original energy/separation criteria.

For each recovered phase, an additional TPD check used log-composition ratios in ${\left[-35,35\right]}^{2}$, the phase itself, 49 deterministic starts, and nested budgets of 50, 100, and 200 random starts. These are declared bounded local searches, not interval bounds or a global certificate. Only the failed record was recomputed; the other 99 phase solutions and the historical training/timing campaign were preserved. All corrected panel metrics and empirical distances were recalculated from the combined records.

For a reference phase $x^{★}$, the sign convention is

(15) $$\mathrm{TPD}\left(w;x^{★}\right)=\sum_{i}w_{i}\left[\ln w_{i}+\ln\gamma_{i}\left(w\right)-\ln x_{i}^{★}-\ln\gamma_{i}\left(x^{★}\right)\right];$$

and negative TPD denotes a first-order instability. The stochastic search was repeated with 18, 50, and 100 Dirichlet starts and SLSQP tolerances $10^{-8}$, $10^{-10}$, and $10^{-12}$, reporting the best value, best-minimum recovery fraction, number of distinct minima, iterations, and evaluations. It remains a local-stochastic diagnostic; deterministic global methods such as GLOPEQ/αBB are required for a continuum certificate [25,26].

No deterministic lower bound for the continuum ternary TPD minimum is computed here. A multistart minimum is an *upper* bound on that minimum, so increasing the number of starts cannot by itself certify stability. Consequently, neither the NRTL numerical reference labels nor the learned ternary phase counts have a global-correctness guarantee. Reported agreement is conditional on the one-/two-phase search, tolerances, and successful convergence. A solver failure is unresolved, not affirmative evidence of a stable single phase. Proposition A7 gives the bound that a future deterministic calculation would have to supply.

### 4.8. Uncertainty, Controls, and Split-Conformal Audit

Here *epistemic uncertainty* denotes uncertainty associated with limited training information and fitted model parameters. Ensemble dispersion is only a proxy for that uncertainty: members can share the same chemical model bias and agree closely while all are wrong. It is distinct from measurement or label noise. *Out-of-distribution* (OOD) means outside the training support, and *abstention* means withholding a prediction and referring the state for additional calculation or measurement.

The temperature-shift ensemble contains K=20 hard potentials with independent data, noise, and initialization seeds. Each member has H=12 and is trained on 290–370 K. Evaluation combines 500 in-domain states (295–365 K) and 500 shifted states (390–450 K). For state *s*,   

(16) $$u_{s}={\left[\frac{1}{N_{c}}\sum_{i}{\mathrm{Var}}_{k=1}^{K}\left(\ln\gamma_{i,s}^{\left(k\right)}\right)\right]}^{1/2}.$$

Pooled and within-domain Spearman correlations receive pair-bootstrap 95% intervals. Negative controls are the distance from the training-temperature interval and fixed-kernel GP uncertainty; high-error detection uses AUROC. A separate 20-member ensemble trained only on $x_{1}\in \left[0.20,0.80\right]$ tests composition shift at the excluded tails.

Selective risk retains the least-uncertain fraction at 17 coverages from 100% to 20%. Two hundred random-retention curves define a pointwise 95% control band, and area under the risk–coverage curve (AURC) provides a scalar summary. A disjoint 500-state in-domain calibration set supplies the finite-sample 90% split-conformal radius from the simultaneous score $r_{s}=\max_{i}\left|{\hat{\ln\gamma }}_{i,s}-\ln\gamma_{i,s}\right|$ [27]. Coverage is audited separately in-domain and under shift; no OOD coverage guarantee is claimed.

### 4.9. Process Stress Test, Runtime, and Statistics

Cross-current extraction begins from one mole with composition proportional to (0.85,0.15,0); a fresh extractant-like component is added at solvent/feed molar ratios 0.25, 0.50, and 1.00 for 4, 8, and 12 stages. The water-rich phase advances. Reference and potential use identical SLSQP settings and start budgets, with independent fixed random-start seeds. Outputs are recovery error, maximum mass-balance residual, and solver evaluations.

Timing uses one numerical thread per backend, three discarded warm-ups, and 30 interleaved repeats on the same feed and start budget. Medians and interquartile ranges, total function evaluations, total SLSQP iterations, CPU model, logical-core count, BLAS configuration, and package versions are archived. This pointwise implementation is a controlled implementation benchmark; it is not a claim about compiled/batched inference or warm-start acceleration.

All method-comparison uncertainty is calculated across ten independent training procedures and summarized by median and interquartile range. Paired Wilcoxon tests operate across seeds. State bootstrap is used only for explicitly state-conditional rank summaries. The original four hypotheses were sharpened as follows: H1 can fail if the hard potential does not improve predictive accuracy or consistency relative to matched soft/classical controls; H2 can fail by branch instability under grid refinement or by any phase-count error, especially near the empirical boundary; H3 can fail if ensemble ranking does not exceed domain-distance/random controls within strata; and H4 can fail if recovery error exceeds one percentage point or mass residual exceeds $10^{-10}$. Algorithms 1–6 provide executable pseudocode.
**Algorithm 1:** Leakage-free fitting and seed-level comparison of excess Gibbs and baseline models1Generate mutually disjoint training, validation, and noise-free test states from the declared synthetic reference.2On the validation split only, choose the hard-potential weight λ and the soft Gibbs–Duhem penalty from their frozen candidate grids.3**for** seed s=1,…,10, **do**4    Draw a new training-noise realization and a new parameter initialization.    Minimize Equation (10) by L-BFGS-B using AD; fit the matched direct, soft-GD.5Redlich–Kister, fixed-α NRTL, free-α NRTL, and fixed-kernel Gaussian-process controls on the identical observations.6    Freeze every fit and evaluate the common untouched test set; store seedwise MAE, convergence state, and thermodynamic diagnostics.7**end for**8Report medians and interquartile ranges across seeds; apply paired Wilcoxon tests to the ten seed-level MAEs, never to individual states.

**Algorithm 2:** Thermodynamic property evaluation from one learned scalar potential
1**Input:** *T*, $x\in \mathrm{int}\Delta^{N_{c}-1}$, identity embeddings $\left\{h_{i}\right\}$, and fitted parameters θ.2Set $g_{\theta }^{E}\leftarrow 0$ and $\nabla_{x}g_{\theta }^{E}\leftarrow 0$.3**for each** unordered pair i<j, **do**4    Construct the symmetric feature vector in Equation (5); evaluate $\psi_{\theta }$, $\partial \psi_{\theta }/\partial x$, and $\partial \psi_{\theta }/\partial T$ analytically.5    Accumulate $x_{i}x_{j}\psi_{\theta }$ and its composition and temperature derivatives.6
**end for**
7Compute each $\ln\gamma_{i}$ from Equation (3).8Compute $H^{E}/\left(nR\right)=-T^{2}\partial \left[g_{\theta }^{E}\right]/\partial T$; approach $x_{i}\to 0^{+}$ for $\ln\gamma_{i}^{\infty }$.9**Return:** $g_{\theta }^{E}$, $\nabla_{x}g_{\theta }^{E}$, lnγ, and the requested thermal or dilute-limit properties.


**Algorithm 3:** Grid-refined binary coexistence and differential-consistency certificate
1**for** grid sizes N∈{2600,10,400,41,600} and each declared isotherm $T_{q}$, **do**2    Evaluate $g^{\mathrm{mix}}\left(x_{1},T_{q}\right)$ for the reference and learned potentials on the same uniform open-simplex grid.3    Construct the one-dimensional lower convex hull by monotonically removing every point that violates increasing secant slope.4    Record the endpoints of the largest skipped interval, or “one phase” if no point is skipped.5
**end for**
6Separate left- and right-branch composition errors, invert both monotone branches for temperature error, and verify stability of the metric under grid refinement.7Independently evaluate $\sum_{i}x_{i}\;d\ln\gamma_{i}/dx_{1}$ on N∈{301,601,1201,2401,4801}; estimate the log–log slope in the spacing *h*.8Interpret $O(h^{2})$ decay for potential-derived models as a numerical certificate of an analytic identity, not as a hypothesis test.


**Algorithm 4:** Mass-conserving ternary two-phase flash and multistart TPD audit
1**Input:** feed z, total mixing-energy evaluator $g^{\mathrm{mix}}$, maximum phase count $N_{p}^{\max}=2$, tolerances, and random seed.2Parameterize phase A by $\left(\beta ,x_{1}^{A},x_{2}^{A}\right)$ and eliminate phase B exactly as $x^{B}=\left(z-\beta x^{A}\right)/\left(1-\beta \right)$.3From four deterministic and the declared number of Dirichlet starts, minimize $\beta g^{\mathrm{mix}}\left(x^{A}\right)+\left(1-\beta \right)g^{\mathrm{mix}}\left(x^{B}\right)$ by SLSQP subject to open-simplex bounds.4If no feasible converged candidate exists, preserve the failed record and invoke Algorithm 5; never accept a failed single-feed fallback as a phase classification. If recovery also fails, report unresolved.5Compare the best feasible split with the single-feed energy; accept two phases only when both energy gain and phase separation exceed the declared thresholds.6For the three declared representative phase states $x^{★}$, minimize Equation (15) from the phase itself and 18, 50, and 100 randomized starts at tolerances $10^{-8}$, $10^{-10}$, and $10^{-12}$.7Archive phase count, phase fractions, compositions, energy margin, successful starts, iterations, function evaluations, recovered minima, and mass-balance reconstruction.8Report a confusion matrix and exact Clopper–Pearson interval over the 100-feed one-/two-phase panel, with accuracy stratified by empirical distance to the opposite class. Separately audit agreement among jointly converged cases; neither statistic is a global certificate.


**Algorithm 5:** Trace-feed recovery with feasible component allocations
1**Input:** failed feed, frozen scalar potential and analytic chemical potentials, archived failure record.2Diagnose floor infeasibility: $z_{i}<\epsilon$ is incompatible with $x_{i}^{A},x_{i}^{B}\geq \epsilon$. Do not clip or renormalize the feed to satisfy an artificial floor.3Use Equation (14) with $r_{i}\in \left[10^{-10},1-10^{-10}\right]$; positivity and mass balance hold by construction.4From 24 deterministic and 32 random allocations (seed 947), minimize the extensive energy with L-BFGS-B and analytic gradient $z_{i}\left(m_{i}^{A}-m_{i}^{B}\right)$.5Refine each candidate by bounded least squares of $m_{i}^{A}-m_{i}^{B}=0$; this prevents a tiny feed fraction from suppressing its equilibrium residual.6Accept only positive, feasible candidates with chemical-potential residual $\leq 10^{-8}$, balance residual $\leq 10^{-12}$, energy decrease $>2\times 10^{-8}$, and phase separation $>2\times 10^{-3}$.7Select the lowest accepted energy and perform the declared log-composition TPD searches from both phases with 50, 100, and 200 nested random starts; reject any observed TPD below $-10^{-8}$.8Preserve the original records; archive the correction separately with input hashes, compositions, residuals, and all start outcomes. Recompute the panel summaries and Figure 9; retain the absence of a continuum global certificate.


**Algorithm 6:** Ensemble ranking, negative controls, split-conformal calibration, and selective risk
1Fit K=20 potentials using independent data/noise/initialization seeds within the declared training domain.2On untouched in-domain and shifted states, compute the ensemble mean, statewise MAE $e_{s}$, and dispersion $u_{s}$ from Equation (16).3Report pooled and within-stratum Spearman correlations with bootstrap intervals; compare against temperature distance and fixed-kernel Gaussian-process uncertainty; compute high-error AUROC.4**for** every retained coverage *c*, **do** keep the *c* least-uncertain states and calculate accepted-state risk; repeat random retention 200 times to obtain a 95% control band.5On a disjoint calibration set, calculate simultaneous residual scores $r_{s}=\max_{i}\left|{\hat{\ell }}_{i,s}-\ell_{i,s}\right|$ and the finite-sample 90% split-conformal quantile.6Audit coverage separately in-domain and under temperature shift; repeat the ranking experiment for a composition-domain shift.7Archive member parameters, predictions, uncertainty, errors, calibration radius, risk–coverage arrays, AURC, and controls.


### 4.10. Algorithms and Executable Pseudocode

The following pseudocodes specify the numerical procedures actually executed by the accompanying scripts. They distinguish architectural identities from finite numerical certificates and expose the randomization, model-selection, equilibrium, and calibration steps needed to reproduce the reported estimands. The symbols AD and SLSQP denote automatic differentiation and sequential least-squares quadratic programming, respectively.

## 5. Conclusions

This study establishes a bounded result: representing one excess Gibbs potential gives a neural surrogate exact resident pure limits, joint permutation equivariance, and analytic Gibbs–Duhem consistency, and those properties propagate through mass-conserving phase calculations. The reviewer-driven campaign replaced saturated demonstrations with ten-seed baselines, numerical refinement studies, thermal and dilute-limit derivatives, 100-feed type-I and type-II phase panels, multistart stability saturation, uncertainty calibration controls, and 30-repeat timing.

The evidence does not support neural superiority. Redlich–Kister matched or exceeded the constrained network, and refitted NRTL dominated on its own synthetic family. The phase classifier made two type-I false-negative decisions relative to the numerical reference. An infeasible numerical composition floor caused one joint solver failure; a mass-conserving allocation reformulation recovered that feed as two-phase coexistence in both models, giving 100 converged records without establishing a global certificate. Pooled ensemble ranking was largely a domain-separation effect; a temperature–distance heuristic was stronger, and conformal coverage collapsed under shift. The pointwise learned flash remained slower despite requiring fewer solver evaluations. These negative outcomes define the method more rigorously than the original favorable aggregates.

A shared molecular encoder preserves the structural results only under the stated symmetry, regularity, and differentiation conditions; the new interface tests do not constitute molecular validation. No chemical-family OOD performance, accuracy for associating or electrolyte systems or critical regions, continuum ternary phase-count certificate, or equal-accuracy acceleration have been demonstrated. Those claims remain outside the present evidence.

The defensible conclusion is therefore structural and methodological: hard free-energy parameterization is preferable to unconstrained neural activity regression when exact thermodynamic relationships are required, but it does not replace classical model selection, external validation, global optimization, or calibrated shift handling. A molecular claim requires licensed experimental VLE/LLE/VLLE data, graph- or descriptor-based identities, component-disjoint evaluation, and comparison with classical and molecular baselines. The supplied fail-closed EXP-6 protocol, raw seed records, pseudocodes, tests, manifests, and generated figures make those next tests executable without pretending they have already been passed. The accompanying code, configurations, data arrays, and numerical records are provided in the Supplementary Materials.

## Figures and Tables

**Figure 1:**
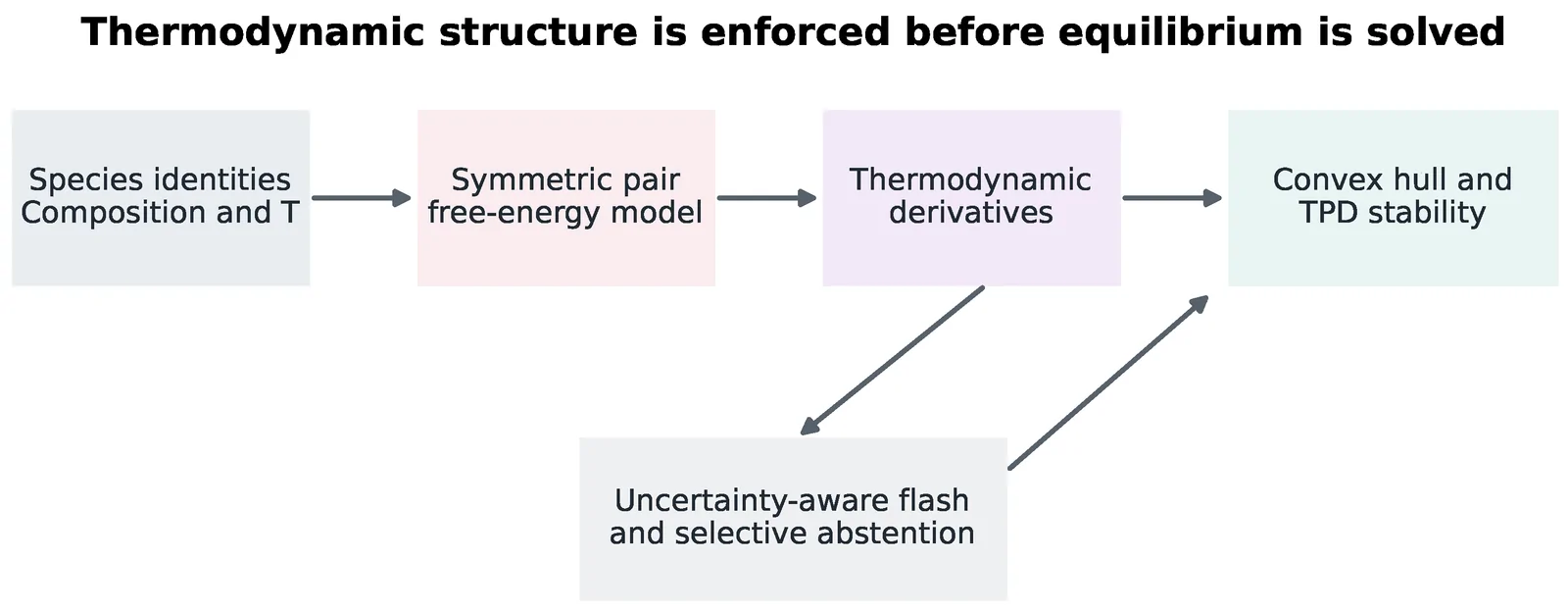
Scientific workflow. A joint species–composition representation defines one invariant excess Gibbs potential. Exact composition derivatives generate activity coefficients and chemical potentials; convexification or tangent-plane-distance (TPD) tests determine phase stability; ensemble dispersion controls abstention. The current pilot executes every block except molecular graph transfer and end-to-end differentiation through the flash.

**Figure 2:**
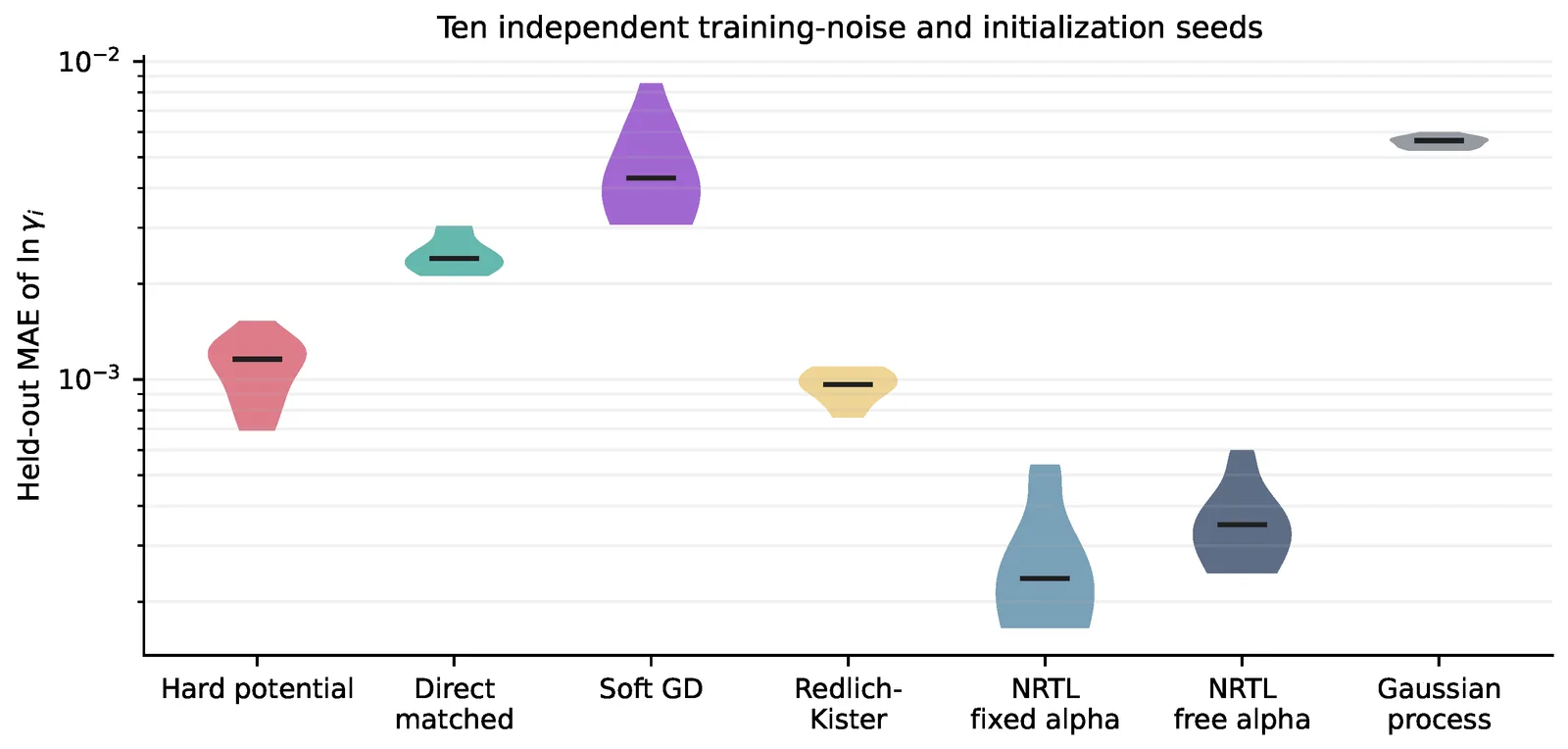
Binary held-out activity-coefficient MAE for ten independent training-noise and initialization seeds. Medians are shown for the hard excess Gibbs potential, matched direct and soft-GD networks, Redlich–Kister, refitted NRTL variants, and a fixed-kernel Gaussian process. The constrained neural model improves on the direct neural controls but not on the correctly specified classical reference family.

**Figure 3:**
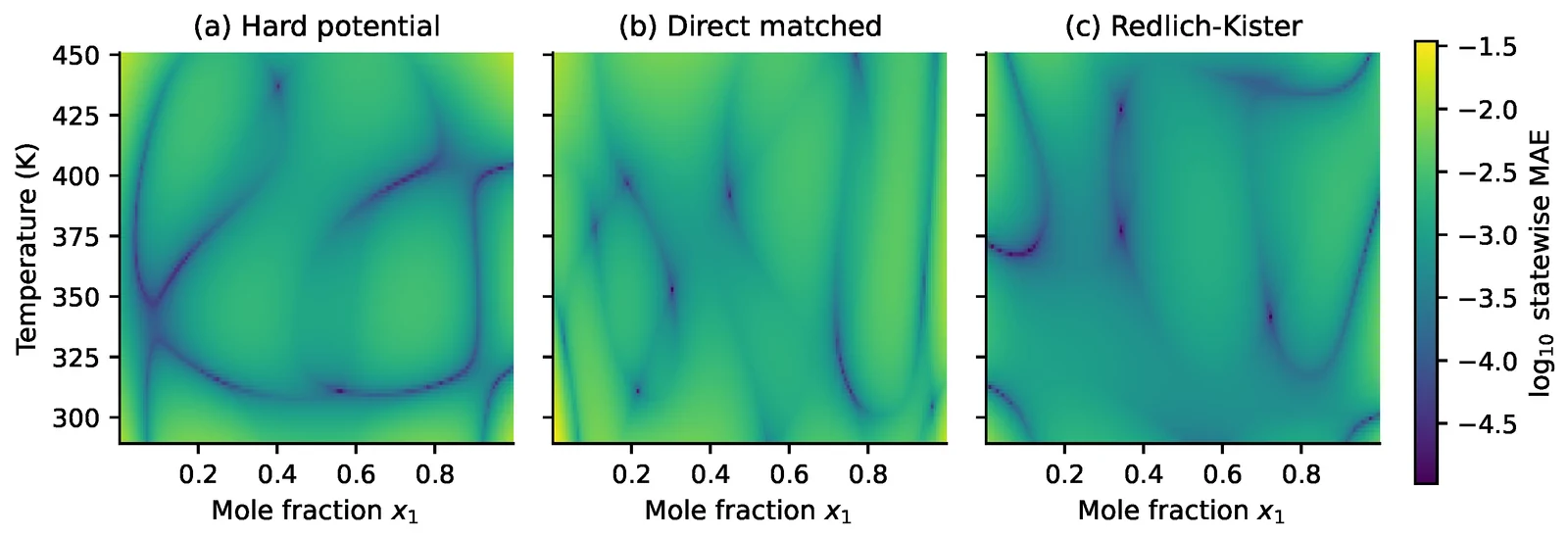
Binary statewise residual maps over composition and temperature: (**a**) hard potential, (**b**) matched direct network, and (**c**) Redlich–Kister. Color denotes $\log_{10}$ of the two-component mean absolute lnγ error.

**Figure 4:**
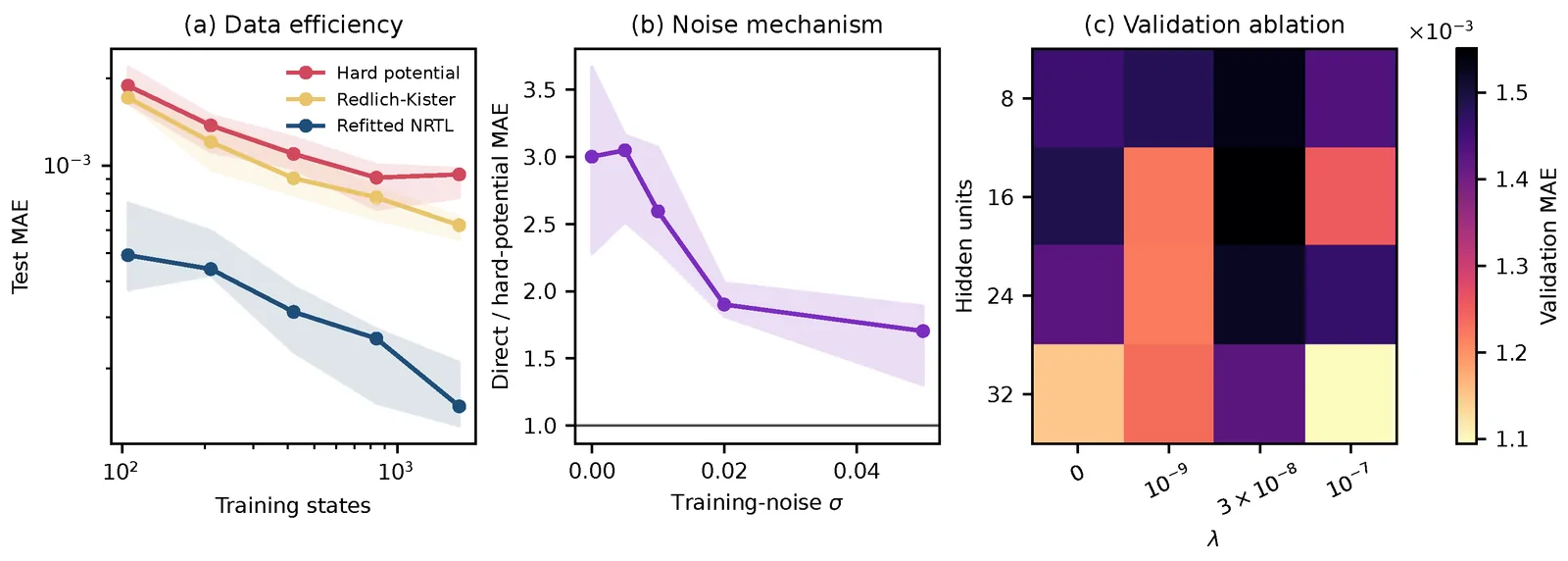
Reviewer-driven robustness campaign. (**a**) Median/IQR test error across ten seeds versus training size. (**b**) Direct-to-hard MAE ratio across noise levels; values above one favor the hard potential. The purple curve shows the median ratio and the shaded band its interquartile range across ten seeds. (**c**) Validation-only ablation of hidden width and regularization.

**Figure 5:**
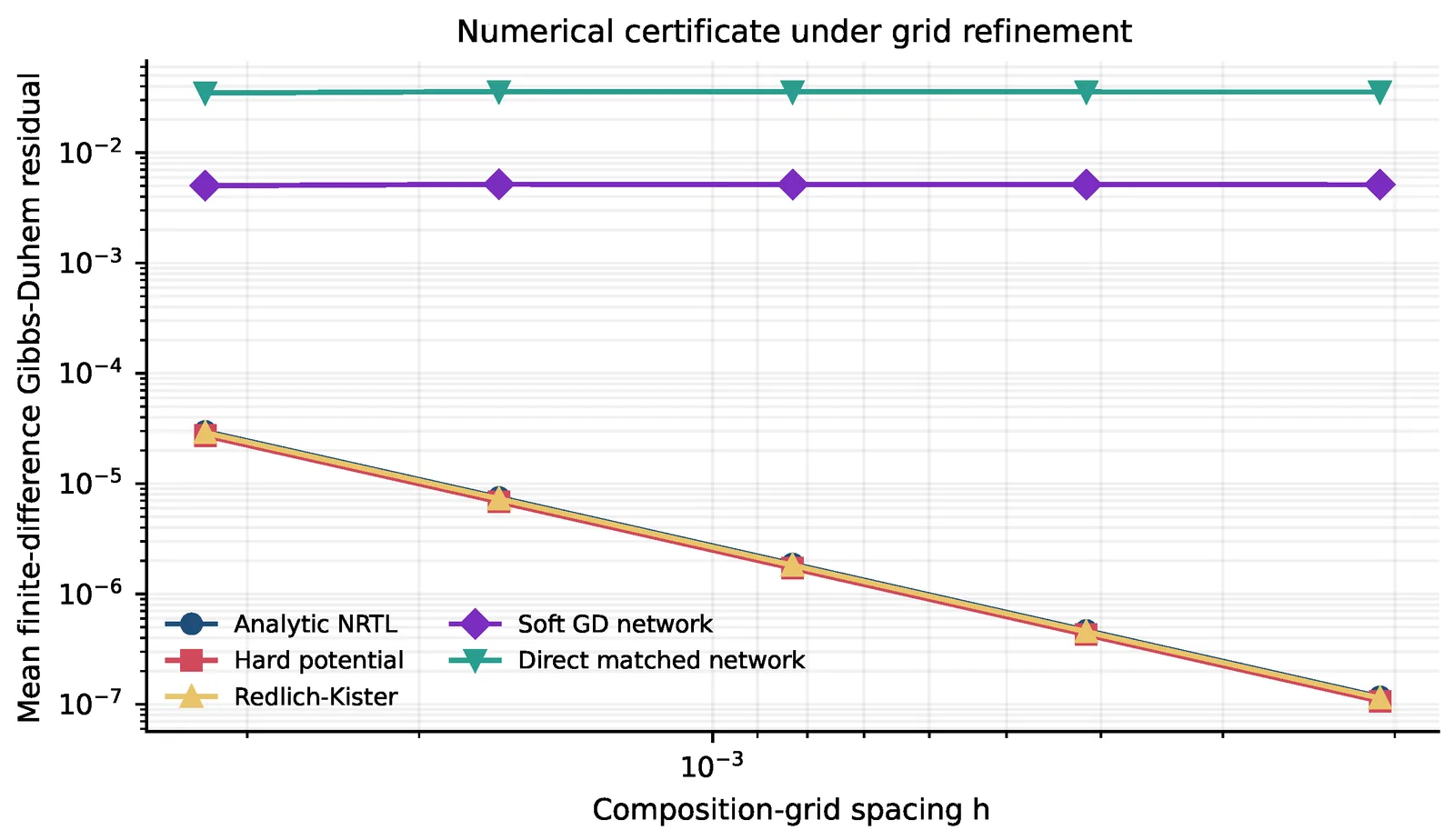
Composition-grid refinement of the mean finite-difference Gibbs–Duhem residual at 300 K. The analytic-reference, hard-potential, and Redlich–Kister curves decay as $O(h^{2})$; direct and soft-penalty networks converge to nonzero residuals.

**Figure 6:**
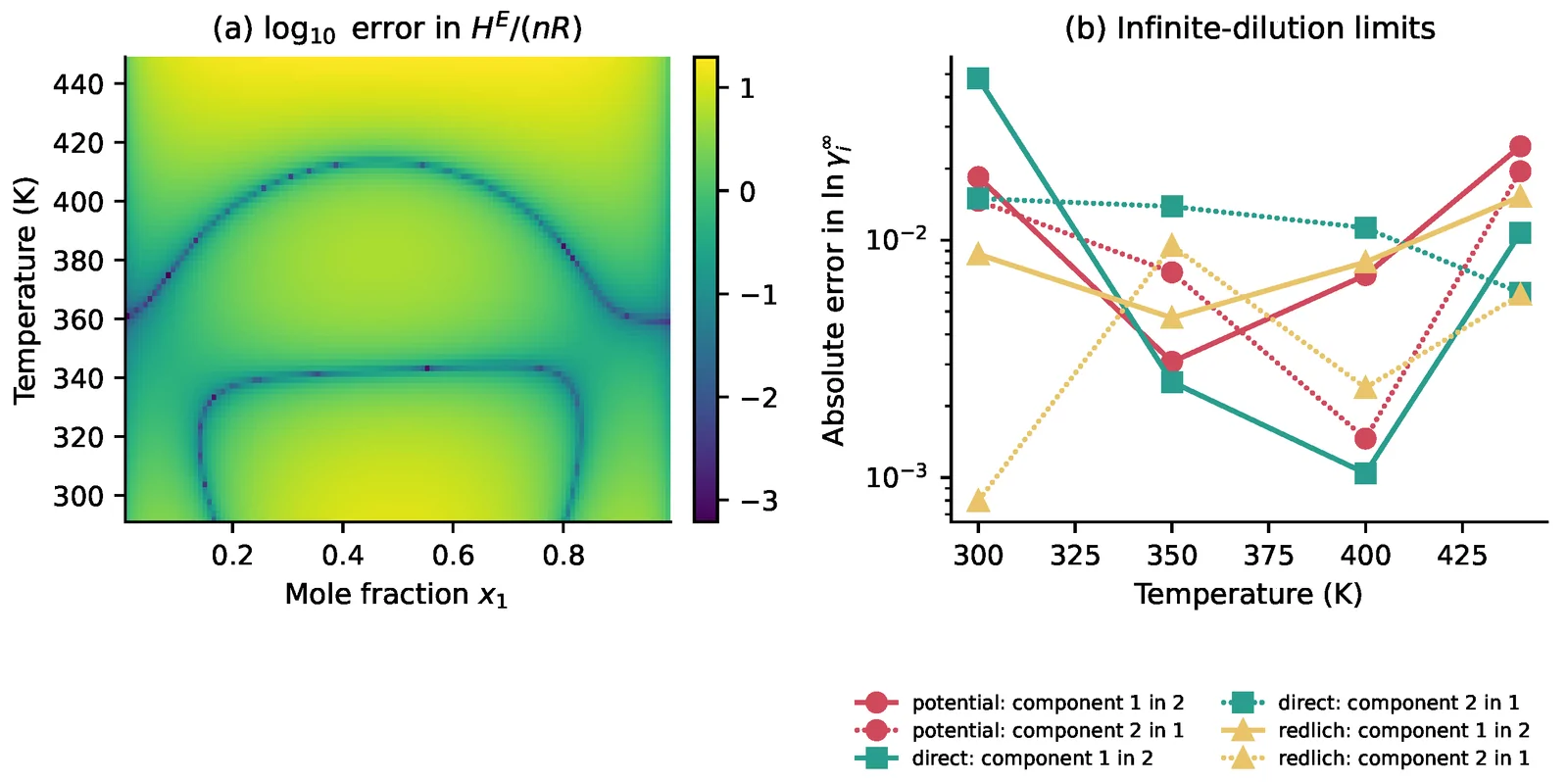
Thermal and dilute-limit diagnostics. (**a**) Error map for $H^{E}/\left(nR\right)=-T^{2}\partial g^{E}/\partial T$. (**b**) Absolute errors of both infinite-dilution directions at four temperatures. Solid curves correspond to component 1 infinitely dilute in component 2; dotted curves correspond to component 2 infinitely dilute in component 1.

**Figure 7:**
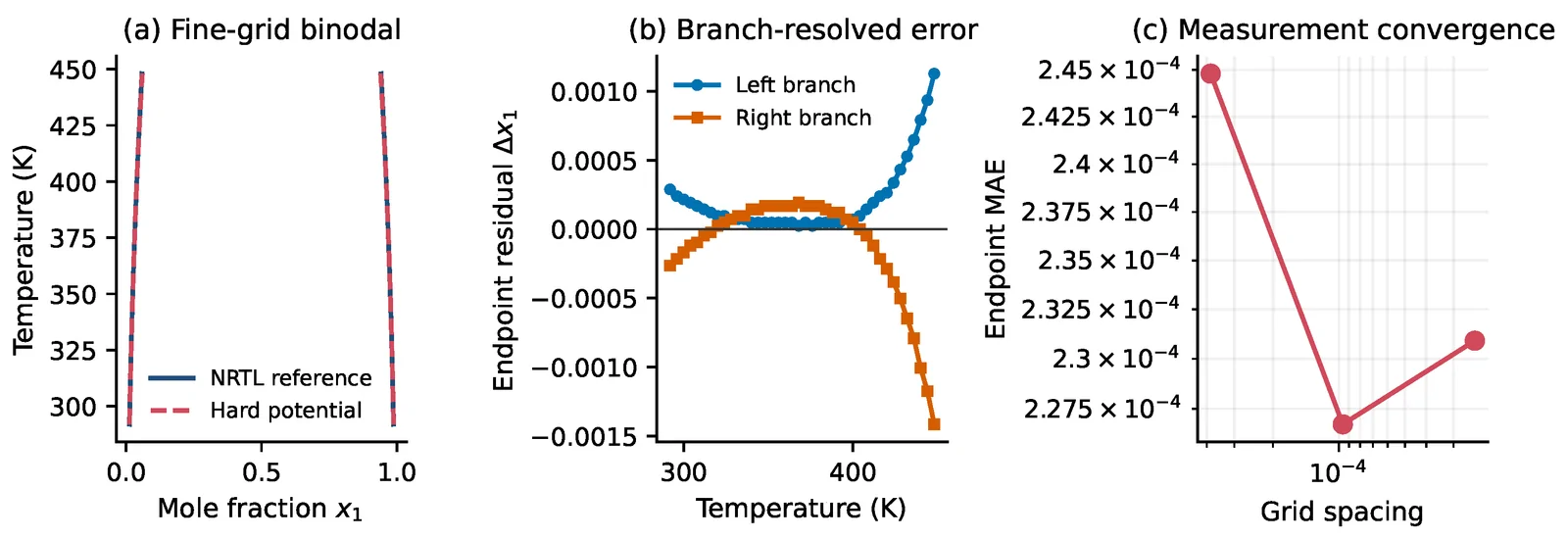
Binary coexistence audit. (**a**) Fine-grid reference and learned binodals; (**b**) signed left/right endpoint residuals versus temperature; (**c**) endpoint MAE under 2600-/10,400-/41,600-point refinement. Composition and inverse-temperature errors are reported separately.

**Figure 8:**
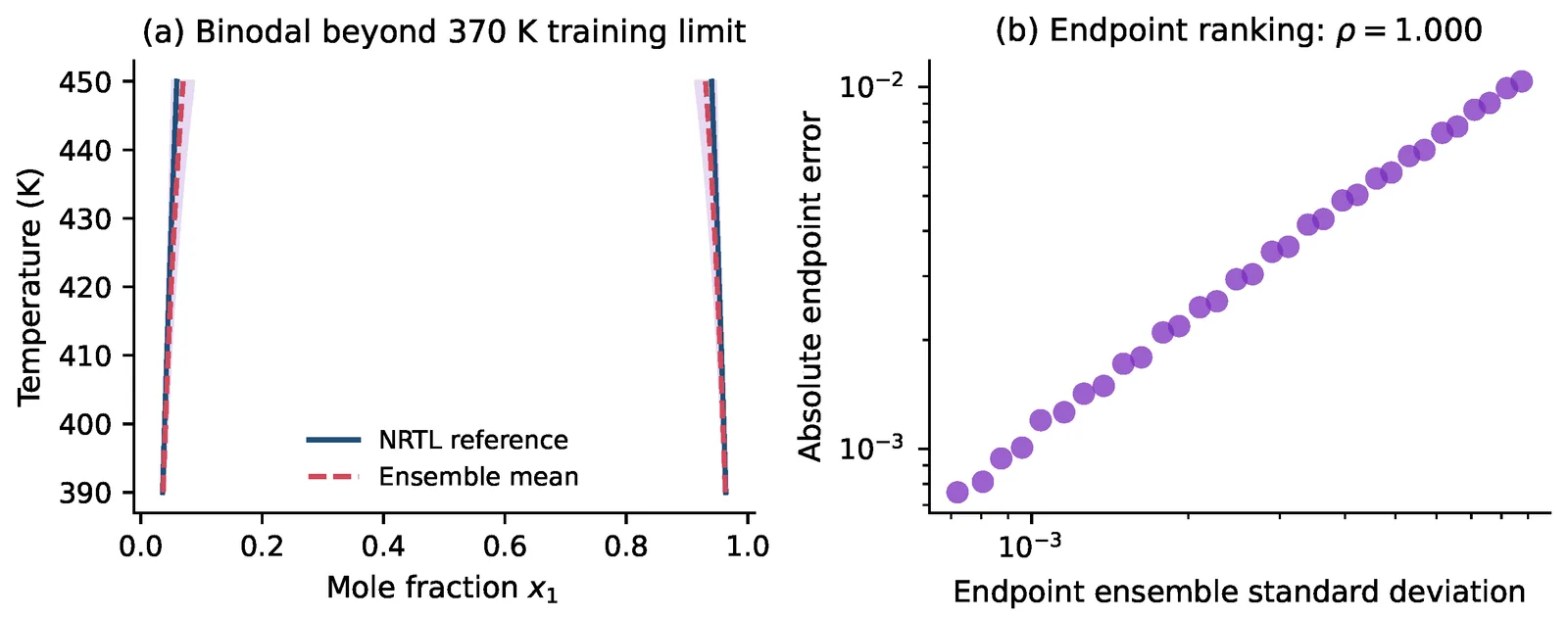
Extrapolative binary coexistence. (**a**) The 20-member mean and the purple shaded nominal ±1.96 ensemble-standard-deviation band beyond the 370 K training limit; (**b**) endpoint error versus endpoint dispersion. The monotone shared dependence on temperature distance precludes a mechanism-independent uncertainty claim.

**Figure 9:**
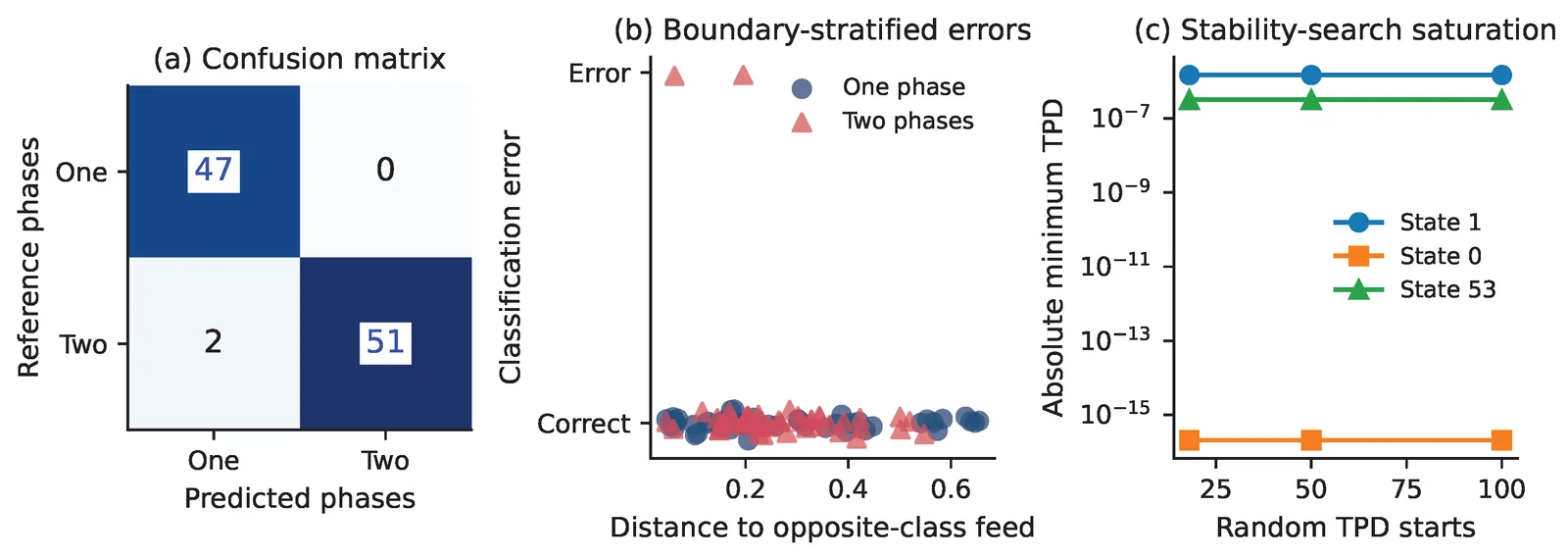
Type-I ternary stress test. (**a**) Reference-row confusion matrix over 100 feeds, including the recovered trace feed; (**b**) errors stratified by empirical distance to the nearest opposite-class feed using corrected labels; (**c**) archived multistart TPD minimum for representative states as the random-start budget increases. The separate recovered-state checks are summarized in Table 2.

**Figure 10:**
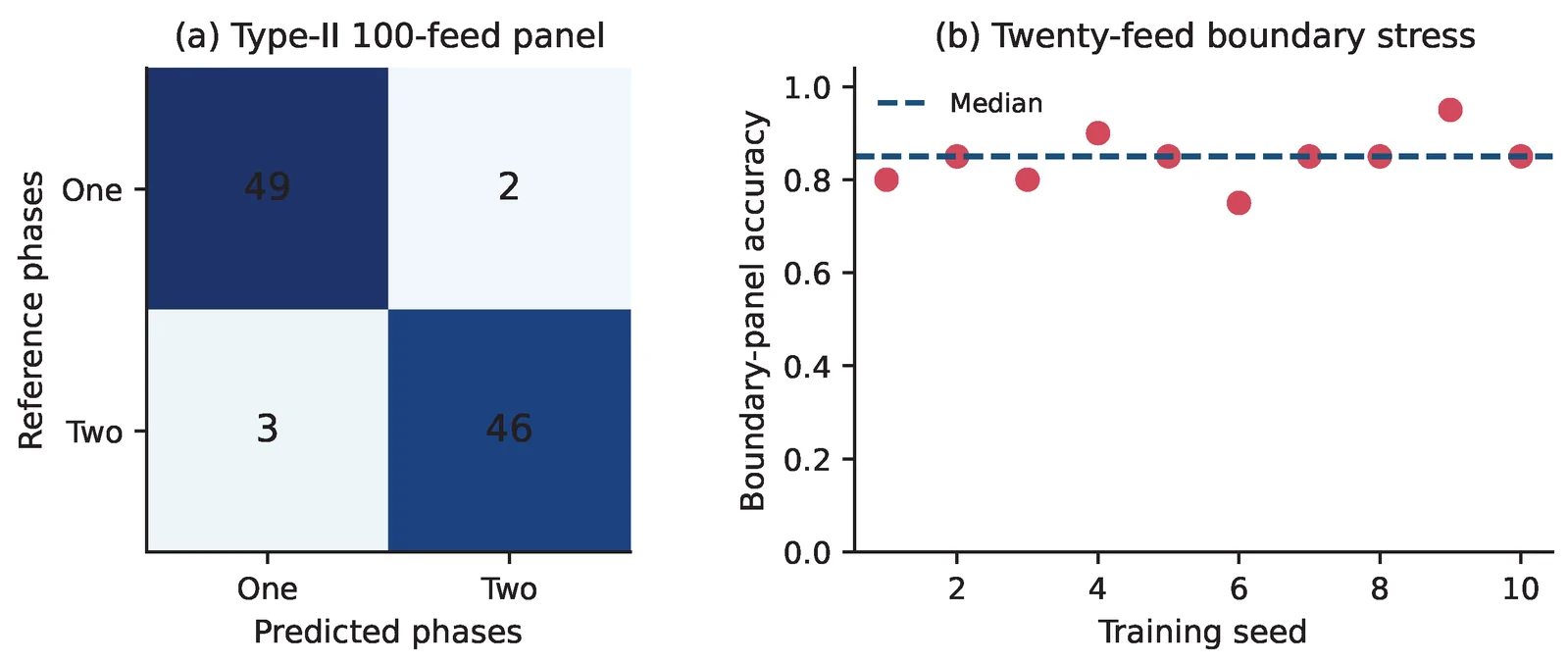
Supplemental type-II synthetic control. (**a**) One-/two-phase confusion matrix for 100 feeds; (**b**) near-boundary phase accuracy across ten training seeds.

**Figure 11:**
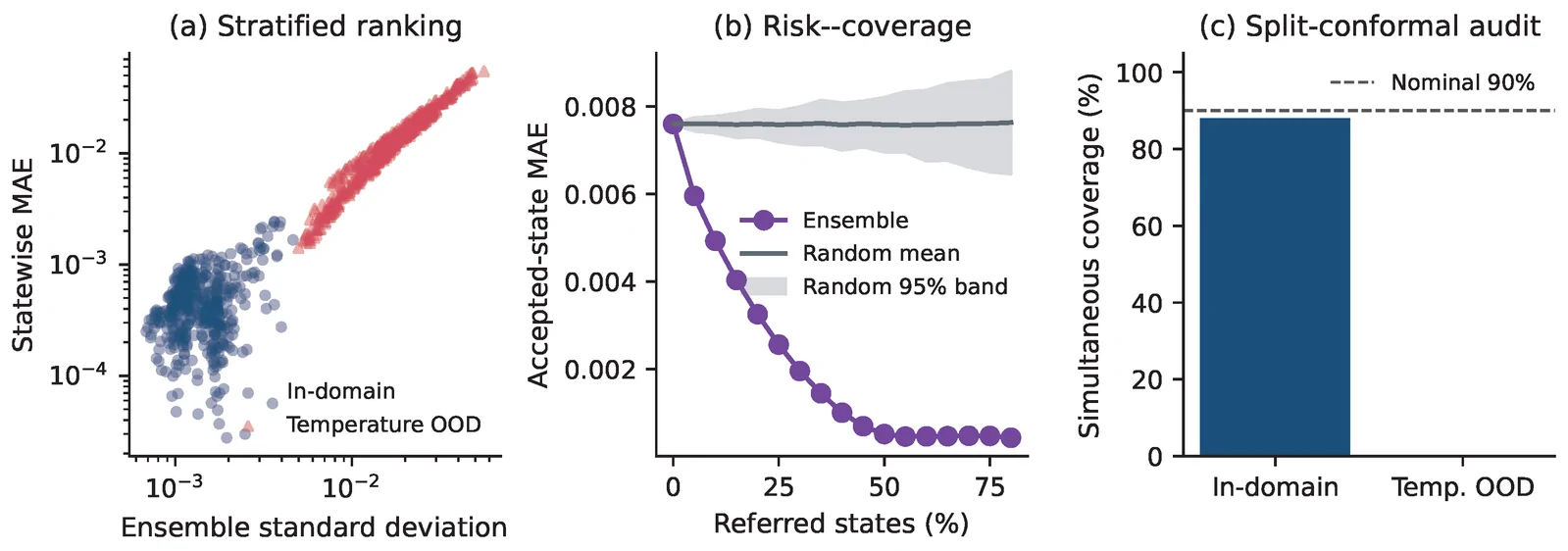
Uncertainty audit. (**a**) Error versus 20-member ensemble dispersion, separated by domain; (**b**) risk–referral curve with a 95% band from 200 random controls; (**c**) simultaneous 90% split-conformal coverage in-domain and under temperature shift.

**Figure 12:**
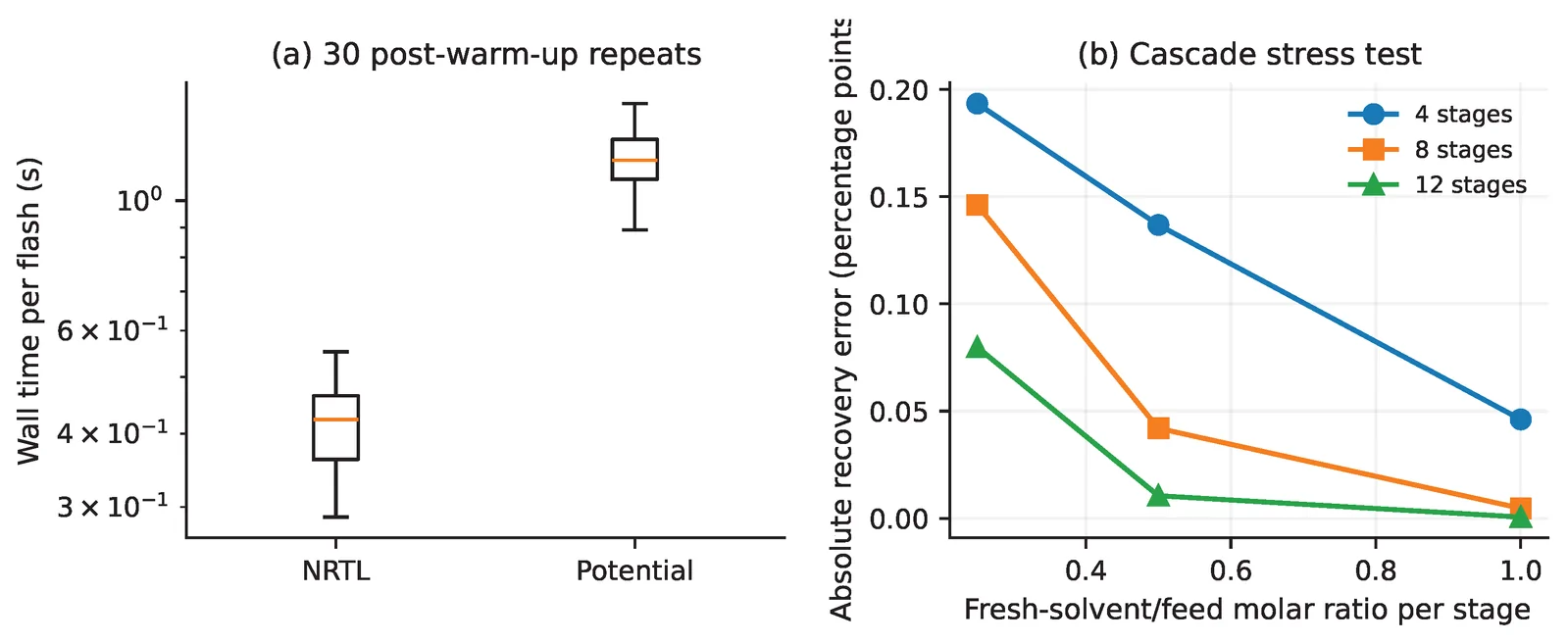
Controlled performance audit. (**a**) Post-warm-up wall time for 30 matched flashes; (**b**) recovery error across solvent/feed ratios and cascade lengths.

**Table 1:** Seed-0 thermodynamic property audit for every binary baseline. Direct-output models use the explicitly labeled operational reconstruction $g^{E}=\sum_{i}x_{i}\ln\gamma_{i}$ for the hull; this does not restore integrability.

Method	$\ln\gamma^{\infty }$ MAE	GD Residual (N=4801)	Binodal Endpoint MAE
Hard potential	$1.20\times 10^{-2}$	$1.07\times 10^{-7}$	$2.50\times 10^{-4}$
Direct matched	$1.35\times 10^{-2}$	$3.55\times 10^{-2}$	$3.72\times 10^{-3}$
Soft GD	$2.64\times 10^{-2}$	$5.12\times 10^{-3}$	$3.26\times 10^{-4}$
Redlich–Kister	$6.91\times 10^{-3}$	$1.14\times 10^{-7}$	$1.51\times 10^{-4}$
NRTL, fixed α	$8.04\times 10^{-4}$	$1.17\times 10^{-7}$	$3.60\times 10^{-5}$
NRTL, free α	$2.17\times 10^{-3}$	$1.15\times 10^{-7}$	$7.68\times 10^{-5}$
Gaussian process	$5.45\times 10^{-2}$	$1.29\times 10^{-1}$	$1.03\times 10^{-2}$
UNIQUAC	$2.45\times 10^{-1}$	$1.75\times 10^{-7}$	$7.90\times 10^{-3}$

**Table 2:** Corrective rerun of the trace-component feed at index 47. Dimensionless chemical potentials are $m_{i}=\ln x_{i}+\ln\gamma_{i}$; Δg is the decrease from the single-feed energy. Full phase compositions and per-start records are archived.

Diagnostic	NRTL Reference	Learned Potential
Phase fraction β	0.658128547	0.658146626
Energy decrease Δg	0.355200294	0.355352092
$\max_{i}\left|m_{i}^{A}-m_{i}^{B}\right|$	$5.87\times 10^{-15}$	$1.78\times 10^{-15}$
Maximum component-balance residual	$1.11\times 10^{-16}$	$1.11\times 10^{-16}$
Smallest observed TPD	$-1.34\times 10^{-16}$	$-9.96\times 10^{-16}$
Accepted two-phase starts/total	54/56	54/56

**Table 3:** Thermodynamic contract and exact scope of each implementation statement.

ID	Requirement	Mathematical Statement	Status in This Study
A1	Regularity	$g_{\theta }^{E}$ is continuous on the closed simplex and smooth in its interior.	Exact for finite tanh parameters.
A2	Extensivity	$G_{\theta }\left(T,\lambda n\right)=\lambda G_{\theta }\left(T,n\right)$.	Exact by molar construction.
A3	Joint permutation	Jointly relabeling identities and compositions leaves $g_{\theta }^{E}$ invariant and permutes lnγ.	Exact; nontrivial ternary test and ordered-feature ablation.
A4	Pure boundary	$g_{\theta }^{E}\left(T,e_{i}\right)=0$ and $\ln\gamma_{i}\left(T,e_{i}\right)=0$.	Exact through $x_{i}x_{j}$ factors.
A5	Phase feasibility	$\beta^{\alpha }\geq 0$, $x^{\alpha }\in \Delta^{N_{c}-1}$, $\sum_{\alpha }\beta^{\alpha }=1$, $\sum_{\alpha }\beta^{\alpha }x^{\alpha }=z$.	Exact flash parameterization.
A6	Stability	The stable state minimizes total Gibbs energy and admits no negative-TPD trial phase.	Global only on the finite binary grid; local-stochastic ternary diagnostic with start/tolerance saturation.
A7	Coordinate objectivity	Activity coefficients are independent of the chosen simplex chart.	Proven in Appendix A.

## Data Availability

All results analyzed in this study are synthetic and are generated by the supplied code. The package includes source code, frozen configurations, generated arrays, fitted parameters with layout metadata, phase-boundary tables, seed-level JSON records, uncertainty arrays, figure scripts, and a SHA-256 manifest. No confidential, proprietary, or third-party experimental dataset was used, and EXP-6 external molecular validation was not executed.

## Supplementary Materials

The following supporting information can be downloaded at https://github.com/luisrojasvaldivia-sudo/ijms-27-08767, accessed on 5 September 2026.

## Abbreviations

The following abbreviations are used in this manuscript:

AURC	Area under the risk–coverage curve
BLAS	Basic Linear Algebra Subprograms
CP	Clopper–Pearson
EoS	Equation of state
GNN	Graph neural network
IJMS	International Journal of Molecular Sciences
LLE	Liquid–liquid equilibrium
MAE	Mean absolute error
NRTL	Non-random two-liquid
OOD	Out of distribution
SciML	Scientific Machine Learning
TPD	Tangent-plane distance
UQ	Uncertainty quantification
VLE	Vapor–liquid equilibrium
VLLE	Vapor–liquid–liquid equilibrium

## Appendix A. Formal Results and Proofs

**Proposition** **A1** (Gibbs–Duhem identity inherited from one extensive potential)**.** *Let $G_{\theta }\left(T,P,n\right)$ be twice continuously differentiable in the positive-mole interior and positively homogeneous of degree one in n. Define $\mu_{i}=\partial G_{\theta }/\partial n_{i}$. At fixed T,P,*

(A1) $$\sum_{i=1}^{N_{c}}n_{i}\;d\mu_{i}=0,\;\sum_{i=1}^{N_{c}}x_{i}\;d\ln\gamma_{i}=0.$$

**Proof.** Euler’s theorem for a degree-one differentiable function gives $G_{\theta }=\sum_{i}n_{i}\mu_{i}$. Differentiating at fixed T,P yields

(A2) $$dG_{\theta }=\sum_{i}\mu_{i}\;dn_{i}+\sum_{i}n_{i}\;d\mu_{i}.$$

The defining total differential is $dG_{\theta }=\sum_{i}\mu_{i}\;dn_{i}$; subtraction gives the first identity. Decomposing $\mu_{i}=\mu_{i}^{\circ }+RT\ln x_{i}+RT\ln\gamma_{i}$, using $\sum_{i}x_{i}\;d\ln x_{i}=\sum_{i}dx_{i}=0$, and dividing by nRT give the second identity.    □

**Proposition** **A2** (Joint permutation invariance and equivariance)**.** *Let the pair map in Equation (4) share parameters across unordered pairs and let its features satisfy*

(A3) $$\phi_{ij}\left(x_{i},h_{i},x_{j},h_{j}\right)=\phi_{ji}\left(x_{j},h_{j},x_{i},h_{i}\right).$$

*Then $g_{\theta }^{E}$ is invariant under every joint permutation of species identities and compositions. Its gradient, and hence lnγ, is equivariant under the same permutation.*

**Proof.** A joint permutation only reindexes the set of unordered pairs. Each summand is unchanged because $x_{i}x_{j}=x_{j}x_{i}$ and the feature vector is symmetric. Therefore, the scalar sum is invariant. Differentiating the identity g(Px,PM)=g(x,M) with respect to x gives ∇g(Px,PM)=P∇g(x,M) for the permutation matrix P. Equation (3) preserves that transformation.    □

**Proposition** **A3** (Encoder-conditional structural preservation)**.** *Let $E_{\eta }$ be a shared species-wise map with atom-permutation-invariant graph output, and let its finite outputs be independent of composition. Use those outputs in Equation (5), retaining the symmetric pair sum and $x_{i}x_{j}$ factors. If the resulting scalar potential is $C^{2}$ in the simplex interior and its first derivatives extend continuously to the pure vertices, it is invariant to atom relabeling and joint species relabeling, its activity vector is species-permutation-equivariant, its resident pure limits are exact, and it satisfies Gibbs–Duhem in the interior.*

**Proof.** An atom relabeling leaves each embedding unchanged by assumption. A joint species permutation maps the matrix of embeddings to PH while mapping x to Px. The feature construction is symmetric in each unordered pair; therefore reindexing the pair sum leaves the scalar unchanged. Differentiating this invariant scalar yields an equivariant gradient and activity vector. At a pure vertex, every pair prefactor vanishes, and the resident gradient cancels in Equation (3). Finally, molar construction gives degree-one extensivity, so the first proposition applies. No step uses a particular message-passing depth or learned weight value. Conversely, a species-position-dependent encoder, order-sensitive graph readout, singular boundary embedding, or omitted composition chain rule violates the stated hypotheses; the proof is not architecture-unconditional.    □

**Proposition** **A4** (Exact resident pure-component limit)**.** *For Equation (4), $g_{\theta }^{E}\left(e_{k}\right)=0$ and $\ln\gamma_{k}\left(e_{k}\right)=0$ for every component k and all finite parameter values.*

**Proof.** At $x=e_{k}$, every product $x_{i}x_{j}$ with i<j is zero; hence $g_{\theta }^{E}=0$. In Equation (3), the weighted gradient is $\partial g_{\theta }^{E}/\partial x_{k}$ because $x_{k}=1$ and all other mole fractions vanish. For the resident component, the two gradient terms therefore cancel exactly.    □

**Proposition** **A5** (Finite non-resident infinite-dilution limit)**.** *For a component i diluted into pure component k≠i, Equation (4) has the one-sided limit*

(A4) $$\lim_{x\to e_{k},\;x_{i}>0}\ln\gamma_{i}=\psi_{\theta }\;\left(\phi_{ik}\left(T,e_{k},M\right)\right),$$

*provided the pair map and its first composition derivatives extend continuously to that vertex, as they do for the stated finite-parameter tanh architecture. Thus the resident limit is fixed by the boundary factor, whereas the non-resident limit remains identifiable from data through the pair map.*

**Proof.** At $e_{k}$, $g_{\theta }^{E}=0$ and $\partial g_{\theta }^{E}/\partial x_{k}=0$. In the derivative with respect to the absent component $x_{i}$, every pair contribution vanishes except (i,k): differentiating $x_{i}x_{k}\psi_{\theta }$ and taking $x_{i}\to 0^{+}$, $x_{k}\to 1^{-}$ gives $\psi_{\theta }\left(\phi_{ik}\right)$. Substitution in Equation (3) yields Equation (A4).    □

**Remark** **A1** (Independence of the simplex chart)**.** *Let two continuously differentiable ambient extensions agree on the simplex. Their gradient difference is orthogonal to every tangent direction and is therefore a scalar multiple a1 of the all-ones normal. The linear operator in Equation (3) annihilates that ambiguity:*

(A5) $${\left[a1-1\sum_{j}x_{j}a\right]}_{i}=a\left(1-\sum_{j}x_{j}\right)=0.$$

*Consequently, the computed activity coefficients do not depend on which differentiable ambient extension or independent coordinate chart is used, provided all evaluations refer to the same simplex.*

**Proposition** **A6** (Equality of chemical potentials in active phases)**.** *Consider an interior regular solution of Equation (13) with $\beta^{\alpha }>0$ for each active phase. Then, for every component present and every pair of active phases α,κ, $\mu_{i}^{\alpha }=\mu_{i}^{\kappa }$.*

**Proof.** Write the total phase mole vectors as $n^{\alpha }$ and enforce $\sum_{\alpha }n^{\alpha }=n^{\mathrm{feed}}$ using component multipliers $\lambda_{i}$. Stationarity of the Lagrangian with respect to every interior $n_{i}^{\alpha }$ gives $\partial G^{\alpha }/\partial n_{i}^{\alpha }=\lambda_{i}$. The left-hand side is $\mu_{i}^{\alpha }$, so all active phases share the same $\lambda_{i}$.    □

**Proposition** **A7** (A continuum TPD lower bound and an energy-gap certificate)**.** *Fix T,P and a feed z. Let $g=g^{\mathrm{mix}}$ be the dimensionless molar Gibbs energy, and let m be any candidate dimensionless chemical-potential vector after removal of pure-species standard terms. Suppose a certified number L satisfies*

(A6) $$L\leq \underset{w\in \Delta^{N_{c}-1}}{\inf}\left[g(w)-m\cdot w\right].$$

*For a feasible candidate phase assemblage of energy $G_{c}=\sum_{\alpha }\beta^{\alpha }g\left(x^{\alpha }\right)$, the unrestricted convexified minimum $G_{*}$ obeys*

(A7)
$$0\leq G_{c}-G_{*}\leq G_{c}-m\cdot z-L.$$

*If all active phases share exactly $m=\ln x^{\alpha }+\ln\gamma \left(x^{\alpha }\right)$, then $G_{c}=m\cdot z$ and the bound is −L. In particular, a proven nonnegative TPD everywhere yields a global supporting plane and global optimality within the specified thermodynamic model.*

**Proof.** For any admissible assemblage, Equation (A6) implies $g\left(w^{\alpha }\right)\geq m\cdot w^{\alpha }+L$. Multiplying by its nonnegative phase fractions and using their unit sum and material balance yields G≥m·z+L. Taking the infimum gives the lower bound on $G_{*}$. Feasibility gives $G_{*}\leq G_{c}$, proving Equation (A7). Under exact common chemical potentials, Euler’s identity gives $g\left(x^{\alpha }\right)=m\cdot x^{\alpha }$ for every active phase. If a residual $\delta \geq |G_{c}-m\cdot z|$ is used instead, the conservative gap bound is δ−L.    □

**Remark** **A2** (What the implemented searches do not certify)**.** *The distinction is between nonnegative TPD* for every composition*, which supplies a global supporting-plane certificate, and nonnegative values at finitely many local minimizers, which do not. A local minimum cannot replace L in Equation (A6). No such L is produced by the present ternary solver. Even a zero energy-gap bound does not establish a unique phase count at degenerate coexistence states. The binary hull is global only on its finite grid; the reported ternary decisions remain conditional numerical estimates.*

### Analytic NRTL Composition Derivative

For reproducibility, define $D_{i}=\sum_{k}x_{k}G_{ki}$ and $N_{i}=\sum_{j}x_{j}\tau_{ji}G_{ji}$. Equation (9) is $g^{E}=\sum_{i}x_{i}N_{i}/D_{i}$. Its ambient derivative is

(A8) $$\frac{\partial g^{E}}{\partial x_{m}}=\frac{N_{m}}{D_{m}}+\sum_{i}x_{i}G_{mi}\frac{\tau_{mi}D_{i}-N_{i}}{D_{i}^{2}}.$$

Substitution of Equation (A8) into Equation (3) generated all reference activity coefficients. No finite-difference derivative was used for training or reference labels.

## Appendix B. Reproducibility Protocol and Artifact Manifest

### Appendix B.1. Exact Execution Sequence

The complete reviewer-driven sequence from the project root is:

python3 -m pip install -r requirements.txt
python3 tests/test_thermo.py
python3 experiments/run_all.py
python3 experiments/run_major_revision.py
python3 experiments/run_type_ii_control.py
python3 experiments/run_extrapolative_binodal.py
latexmk -pdf -interaction=nonstopmode -halt-on-error ijms-27-08767-done.tex

The first orchestrator preserves the originally reviewed campaign; the second produces all revised baselines, refinement, derivative, phase, uncertainty, and timing records; the third supplies the independent type-II topology control; and the fourth evaluates an extrapolative temperature split without retraining. Generated LaTeX macros are imported directly by the manuscript. The external validation runner is intentionally excluded because it must stop until cited experimental data and a completed provenance manifest exist.

### Appendix B.2. Environment and Controlled Timing

The revised campaign ran on Linux 6.18.35, Python 3.12.13, and an Intel Xeon Platinum 8272CL at 2.60 GHz with nine logical CPUs visible to the container. Timing fixed OMP_NUM_THREADS, OPENBLAS_NUM_THREADS, MKL_NUM_THREADS, and NUMEXPR_NUM_THREADS to one. NumPy 2.3.5 used SciPy OpenBLAS 0.3.30 (64-bit integers, dynamic Haswell target); SciPy 1.17.0, scikit-learn 1.8.0, Matplotlib 3.10.8, and Autograd 1.8.0 were recorded through package metadata. The complete numpy.show_config() output is embedded in results/revision_metrics.json. The revision campaign consumed 793.343 s on that allocation; the type-II control is timed separately. Bitwise equality is not promised across BLAS, CPU, or optimizer versions, but seeds, tolerances, and estimands are fixed.

### Appendix B.3. Model Reconstruction Metadata

For the pair network, a flat parameter vector is decoded in the exact order

(A9) $$[{\mathrm{vec}}_{C}\left(W\right),b,a,c],$$

where $W\in R^{H\times F}$ is row-major. For three-dimensional one-hot embeddings, F=12. Binary principal models use H=16, $T_{c}=370$ K, and $T_{s}=80$ K; ternary models use H=20, $T_{c}=323.15$ K, and $T_{s}=50$ K; and uncertainty members use H=12, $T_{c}=370$ K, and $T_{s}=80$ K. The embedding matrix and hidden-unit count are stored with every model NPZ; configs/pilot.json freezes all split sizes, selection grids, flash thresholds, and start/tolerance budgets.

The binary train/validation/test data seeds are 42/43/99. Seed ranges for training replicates, noise sweeps, ensembles, flash starts, bootstrap samples, and timing are named at their point of use in the scripts and written into the raw records. Randomness is generated by NumPy’s default_rng; scikit-learn estimators receive explicit random_state values.

### Appendix B.4. Automated Tests and Numerical Failure Conditions

The permanent tests verify: (i) analytic NRTL Gibbs–Duhem behavior against a refined finite-difference diagnostic; (ii) exact resident pure limits for arbitrary neural parameters; (iii) nontrivial ternary joint-permutation equivariance; (iv) agreement of the analytic neural temperature derivative with a centered finite difference; and (v) independently fixed NRTL infinite-dilution values at four temperatures. Experiment scripts fail if the binary binodal disappears from too many isotherms, a balanced ternary phase panel cannot be formed, experimental provenance is absent, a component-disjoint split leaks an InChIKey, or a supplied data digest differs from its frozen manifest.

Four additional tests in tests/test_review_round2.py exercise the proposed encoder interface using untrained abstract graphs: atom-relabeling invariance of a shared sum-readout encoder, all six ternary species permutations with dense embeddings, resident pure limits, and analytic composition derivatives against centered differences. They test the algebraic interface only; the fixtures are not chemical molecules and supply no chemical-transfer evidence. The same script audits convergence flags in the archived phase records without refitting or deleting failed cases. Run python3 tests/test_review_round2.py; the optional –report argument writes the diagnostics archived in results/review_round2_audit.json. The principal training, uncertainty, and timing metrics remain those of the original archived campaign, not a newly executed molecular experiment.

The separately executed corrective rerun is reproduced by running python3 with experiments/recover_phase_failure.py, followed by scripts/update_recovered_phase_artifacts.py. It retains the original phase records and frozen models, hashes both inputs, and writes results/phase_failure_recovery.json plus a corrected panel. Six regression checks in tests/test_phase_recovery.py verify the original infeasibility, positive mass-conserving allocations, analytic allocation gradients, independently recomputed equilibrium residuals, declared TPD outcomes, and preservation of the other 99 phase solutions. Thus the package has 15 structural/interface/recovery check groups. The two recovered phase labels, confusion counts, tie-line/fraction metrics, and Figure 9 are updated; the historical model-training and runtime estimates are not relabeled as newly measured values.

### Appendix B.5. Archive Structure

The current archive is organized as follows:

- main.tex, references.bib, Definitions/, sections/, and appendices/: the MDPI manuscript, with current additions and replacements in blue.
- src/, experiments/, tests/, and configs/: the historical thermodynamic core and benchmark campaigns, plus the new encoder-interface checks, allocation-based recovery module, corrective experiment, and regression checks.
- data/, results/, and figures/: synthetic datasets, the unexecuted external-data schema, fitted artifacts, raw metrics, and vector/raster figures.
- scripts/, requirements.txt, environment.yml, LICENSE, and MANIFEST.sha256: build/validation tools, dependencies, license, and file digests.

The build script recomputes MANIFEST.sha256 after all outputs are finalized and verifies every listed digest in a clean extraction. A repository DOI is not included because none was supplied or minted; the authors must deposit the immutable release and add its DOI before submission.
